# *Brickworx* builds recurrent RNA and DNA structural motifs into medium- and low-resolution electron-density maps

**DOI:** 10.1107/S1399004715000383

**Published:** 2015-02-26

**Authors:** Grzegorz Chojnowski, Tomasz Waleń, Paweł Piątkowski, Wojciech Potrzebowski, Janusz M. Bujnicki

**Affiliations:** a International Institute of Molecular and Cell Biology, Trojdena 4, 02-109 Warsaw, Poland; b Faculty of Mathematics, Informatics and Mechanics, University of Warsaw, Banacha 2, 02-097 Warsaw, Poland; c Institute of Molecular Biology and Biotechnology, Faculty of Biology, Adam Mickiewicz University, Umultowska 89, 61-614 Poznan, Poland

**Keywords:** *Brickworx*, model building, nucleic acids

## Abstract

*Brickworx* is a computer program that builds crystal structure models of nucleic acid molecules using recurrent motifs including double-stranded helices. In a first step, the program searches for electron-density peaks that may correspond to phosphate groups; it may also take into account phosphate-group positions provided by the user. Subsequently, comparing the three-dimensional patterns of the P atoms with a database of nucleic acid fragments, it finds the matching positions of the double-stranded helical motifs (A-RNA or B-DNA) in the unit cell. If the target structure is RNA, the helical fragments are further extended with recurrent RNA motifs from a fragment library that contains single-stranded segments. Finally, the matched motifs are merged and refined in real space to find the most likely conformations, including a fit of the sequence to the electron-density map. The *Brickworx* program is available for download and as a web server at http://iimcb.genesilico.pl/brickworx.

## Abstract 2

A computer program that builds crystal structure models of nucleic acid molecules is presented. It can be accessed at http://iimcb.genesilico.pl/brickworx.

## 1. Introduction   

The number of experimentally determined structures of nucleic acid molecules, including nucleic acid–protein complexes, is increasing rapidly in line with recent discoveries and growing interest in the biological functions exerted by nucleic acids beyond their protein-coding capacity. In particular, noncoding RNAs (ncRNAs) have been found to be involved in many cellular processes ranging from the regulation of gene transcription to the catalysis of chemical reactions (Cech & Steitz, 2014 ▶). Many of the ncRNAs that have been structurally characterized form compact, functional, three-dimensional structures that determine their function, in a similar manner to the sequence–structure–function relationships that have been studied for proteins for decades (Doudna, 2000 ▶).

In general, the method of choice for studies of macromolecular structures is X-ray crystallography (Ke & Doudna, 2004 ▶). However, nucleic acid crystallography, unlike protein crystallography, still lacks sufficient methodology to facilitate a straightforward crystal structure-determination process. In particular, computational tools that automatically build a crystal structure model into an experimental electron-density map are markedly less developed for nucleic acids than for proteins. A key step in the determination of a final crystal structure model is interpretation of the electron-density map. The manual procedure is often time-consuming and error-prone, since the crystallographer has to visually interpret the features of a three-dimensional electron density in terms of an atomic model. Automated model-building computer programs can speed up the structure-determination process considerably and help to minimize the amount of errors in modelling (Hattne & Lamzin, 2008 ▶). This is particularly important in the case of crystals containing nucleic acids. Such crystals often diffract poorly, which makes the corresponding electron-density maps very difficult to interpret visually. Currently, several freely available programs exist that can be used for building initial models of nucleic acid structures. These include *Nautilus* (Winn *et al.*, 2011 ▶; Cowtan, 2012 ▶), the *ARP*/*wARP* DNA/RNA model-building module (Hattne & Lamzin, 2008 ▶), *phenix.find_helices_strands* and *phenix.build_rna_helices* (Terwilliger, 2010 ▶), operating exclusively in real space. There are also programs available for iterative model building involving refinement: *LAFIRE* (Yamashita *et al.*, 2013 ▶) and *phenix.autobuild* (Terwilliger *et al.*, 2008 ▶). A few methods have been also developed to facilitate manual model building, such as *RCrane* (Keating & Pyle, 2010 ▶) and *Coot* (Emsley *et al.*, 2010 ▶)

*Nautilus* and *ARP*/*wARP* need to determine both the phosphate and base or sugar ring positions to accurately assign the backbone conformers of a single-stranded polynucleotide fragment of a crystal structure model. However, the detection of bases is in general far more difficult than that of phosphates, in particular at low resolution (Hattne & Lamzin, 2008 ▶; Gruene & Sheldrick, 2011 ▶). In contrast, *phenix.find_helices_strands* and *phenix.build_rna_helices* use a convolution search to find places in the asymmetric unit where an A-RNA or B-DNA helix can be placed (the latter program exclusively builds RNA models). This approach gives reasonable results at low resolution with low-quality maps. However, the available implementations can solely build regular double-stranded models.

In this work, a new method that builds large recurrent nucleic acid motifs (including double-stranded helices) into electron-density maps is described. Unlike other available methods, in our approach if only a fraction of the phosphate-group positions can be detected then a correctly placed complete motif can be built into the electron-density map.

## 2. Materials and methods   

### 2.1. Reference structures used as benchmarks   

For training the support vector machine (SVM) classifier, a set of representative crystal structure models of protein–RNA complexes solved at resolutions between 3.0 and 4.0 Å were selected using nonredundant sets of RNA-containing three-dimensional structures (Leontis & Zirbel, 2012 ▶). If diffraction data were not available for a given crystal structure, a structure with experimental data was selected from a corresponding equivalence class. Finally, crystal structure models described as ‘conservatively optimized’ were downloaded from the *PDB_REDO* server (Joosten *et al.*, 2012 ▶) together with the corresponding experimental diffraction data in the binary MTZ format. The set contained 70 structures; the full list of PDB codes is available as Supporting Information.

While the classifier was trained on low-resolution structures, all of the tests were made on a set of crystal structure models solved at medium and high resolution. As a result, during the benchmarks, models built into simulated maps with partially randomized model phases were compared with high-quality reference structures.

The benchmark set of structures was selected using the RCSB PDB (Bernstein *et al.*, 1977 ▶) search service (as of 26 July 2014). Crystal structure models described as ‘conservatively optimized’ together with experimental diffraction data in MTZ format were downloaded from the *PDB_REDO* server (Joosten *et al.*, 2012 ▶). Entries annotated as not reliable by the Uppsala Electron Density Server (Kleywegt *et al.*, 2004 ▶) were removed from the set. Structures without at least two consecutive Watson–Crick base pairs [tested using the 3*DNA* software (Lu & Olson, 2008 ▶) and the RNA Bricks database (Chojnowski *et al.*, 2014 ▶) for DNA and RNA, respectively] and split PDB entries were removed. The final test contained 50 DNA-only structures (randomly selected from 1187), 50 structures of protein–DNA complexes (a random subset of 540 entries), 62 RNA–protein complexes and 31 RNA-only structures. The complete list of test-set structures is available as Supporting Information.

### 2.2. RNA and DNA motif sets   

A-RNA and B-DNA structure models were generated using 3*DNA* (Lu & Olson, 2008 ▶). Coordinates of the RNA recurrent motifs were extracted from the RNA Bricks database (Chojnowski *et al.*, 2014 ▶). As of 8 August 2014 the set contained 2199 RNA fragments, and it can be updated by the users with each new release of the RNA Bricks database. During the benchmarks, however, a set of RNA motifs used for model building was selected separately for each of the tested structures. For a given structure, all motifs derived from structures defined as similar to the query in nonredundant sets of RNA-containing three-dimensional structures (Leontis & Zirbel, 2012 ▶) were excluded.

### 2.3. Simulating low-quality and low-resolution electron-density maps   

The electron-density maps used as benchmarks were generated based on the observed amplitudes, trimmed to a desired resolution, and biased model-derived phases. A procedure for generating phase bias was adapted from the *Computational Crystallography Toolbox* (*cctbx*) library (Grosse-Kunstleve *et al.*, 2002 ▶). It comprises of inversion of a fraction of centric reflection phases and the addition of uniformly distributed random noise to acentric reflection phases. The figures of merit and mean phase differences reported in the results were calculated directly from the biased data.

For each reference structure, maps were generated using structure-factor amplitudes trimmed to 2.5, 3.0, 3.5 and 4.0 Å resolution. Additionally, for each resolution, three sets of biased phases were calculated with a mean phase difference (relative to the values calculated for reference structures) of 18, 35 and 54°, corresponding to figures of merit of 0.92, 0.75 and 0.50, respectively. For each test-set structure, a total of 12 maps of different quality and resolution were generated. For benchmarks, a total of 2316 different electron-density maps were used, which were calculated using 193 reference structures.

### 2.4. Phosphate-detection algorithm   

#### 2.4.1. Electron-density peak searching and parameterization   

For each of the crystal structure models from the training set, a biased phase (*F*_obs_, ϕ_calc_) electron-density map was calculated and normalized. As a result, the standard deviation and mean for all of the maps were 1 and 0, respectively. Next, a peak-search procedure implemented in *cctbx* was performed with a constraint that no two peaks are allowed to be closer than 4.0 Å to each other. Finally, the peaks were parameterized following rules defined in the *Knuspr* program (Gruene & Sheldrick, 2011 ▶). The following parameters were calculated for electron-density map voxels around the peak centre.

(i) The rank-scaled average intensity of voxels within 2.5 Å of the peak centre. Each peak is assigned a score (from 0 to 1) that ranks the peak with respect to the number of peaks that are weaker.
(ii) The correlation coefficient between diametrically opposed map points on a sphere of radius 1.56 Å from the centre of a peak. It should be negative for tetrahedrally shaped peaks.
(iii) (λ_1_ − λ_3_)/λ_2_, where λ_3_ ≥ λ_2_ ≥ λ_1_ ≥ 0 are eigenvalues calculated for the voxel intensities. These are analogous to the principal moments of inertia of a rigid body, and are used to distinguish peaks of tetrahedral symmetry (phosphate groups) from flat objects (*e.g.* bases). The decomposed matrix is a moment-of-inertia tensor calculated for the map voxels within 2.5 Å from the peak centre weighted with corresponding map values.

Finally, the parameters were mapped onto the (0, 1) range to enable the comparison of features derived from different crystals.

#### 2.4.2. Training the support vector machine classifier   

The support vector machine classifier was trained using the set of low-resolution protein–RNA complex structures defined above. Firstly, the strongest peaks were selected in the maps and described using the parameters described in §[Sec sec2.4.1]2.4.1. Next, the peaks were divided into two classes depending on their distance from the P atom of any phosphate group in the reference crystal structure model. Peaks that were found to be closer than 1.5 Å to a P atom were labelled as correct hits, and the remaining peaks were labelled as noise. Initially, an optimal set of the SVM model parameters yielding the largest completeness was determined using a stratified subsampling fivefold cross-validation procedure. The final version of the classifier was trained using an optimal set of the model parameters on a complete training set.

### 2.5. Phosphate-based matching of nucleic acid fragments   

The input to the phosphate-based matching algorithm is a set of putative P atoms (P) and an RNA or DNA three-dimensional motif (M). The task is to find a set of rigid transformations that superpose M onto P with an r.m.s.d. below a given threshold.

Firstly, all possible triplets of P atoms are picked from M and superimposed onto all similar triplets from set P (Supporting Information §S4). The obtained transformations are applied to the complete motif M and the quality of a match is scored by the r.m.s.d. of the closest pairs of P atoms from P and M after superposition. The computational cost of our implementation is reduced by the use of the KD-Trees method as implemented in the Approximate Nearest Neighbor library (http://www.cs.umd.edu/~mount/ANN/). The computational time and the number of plausible solutions grow very rapidly with the size of P and the r.m.s.d. threshold (Supplementary Fig. S37; the time complexity estimates are given in Supporting Information §S4). Therefore, in our implementation, points from P are sequentially removed each time a plausible solution is found, and the r.m.s.d. threshold is gradually increased from 0.5 to 1.0 Å. Additionally, for very large structures (*e.g.* ribosomes), the asymmetric unit is divided into boxes with a number of P atoms of less than 1000 which are processed separately. The computation time of *Brickworx* as a function of the number of detected P atoms is presented in Supplementary Fig. S38.

### 2.6. Model-quality assessment   

All of the crystal structure models built during benchmarks were compared with the corresponding reference structures downloaded from the *PDB_REDO* server. General rules for judging whether a given nucleotide residue was built correctly or not were adapted from Gruene & Sheldrick (2011 ▶). Following the authors’ suggestion, we also introduced a slightly relaxed, base-type independent criterion that should be more appropriate for evaluating models built at low resolution. In total, three distinct validation rules were considered.

(1) *Phosphate positions only.* A putative P-atom position was considered to be correct if the closest P atom from the reference structure (including symmetry mates) was within a distance of 1.5 Å.
(2) *Nucleotide position.* A nucleotide was considered to be correctly placed if both the P and the C1′ atom positions were less than 1.5 and 1.0 Å, respectively, from the corresponding atoms for a nucleotide from the reference structure (or any of its symmetry mates).
(3) *Nucleotide position and base type.* A nucleotide was considered to be correctly built if for a nucleotide in the reference structure (or any of its symmetry mates) (i) the corresponding P atoms were within 1.5 Å distance, (ii) the corresponding C1′ atoms were within a distance of 1.0 Å and (iii) the root-mean-square deviation of atoms common to purines (C1′, C2, C4, C5, C6, N1, N3 and O2) or to pyrimidines (C1′, C2, C4, C5, C6, C8, N1, N3, N7 and N9) was less than 1.0 Å.

The third rule was added because sometimes the programs misassign a base type but otherwise fit the backbone correctly. The strict criterion requires that all three points are met, while the relaxed criterion only requires that the first two be met.

### 2.7. Software used and *Brickworx* implementation   

*Brickworx* and all utility programs were implemented in Python 2.7 and C++ with an extensive use of routines from the *Computational Crystallography Toolbox* (*cctbx*) v.2013_07_05_0005 (Grosse-Kunstleve *et al.*, 2002 ▶), the Approximate Nearest Neighbor library v.1.1 and the LEMON library v.1.3.1 (Dezső *et al.*, 2011 ▶). The SVM classifier was implemented with the use of the *scikit-learn* suite v.0.14.1. The web server interface was developed in the Django framework (http://djangoproject.com) v.1.6. For the benchmarks, the DNA/RNA model-building module from *ARP*/*wARP* v.7.4 patch 2, *Nautilus* v.0.4 and *phenix.find_helices_strands* and *phenix.build_rna_helices* available in *PHENIX* v.1.9-1692 (Adams *et al.*, 2010 ▶) were used.

### 2.8. Experimental electron-density maps   

*Brickworx* was tested on two crystal structure models for which diffraction data with experimental phases had been deposited in the PDB. One model was the group II intron structure (PDB entry 3bwp) solved at 3.1 Å resolution (Toor *et al.*, 2008 ▶). The phases for this structure were determined using Yb^3+^ and iridium hexamine derivatives, resulting in an experimental electron-density map of high quality. The second structure used for testing was a lysine riboswitch (PDB entry 3d0u) solved at 2.8 Å resolution (Garst *et al.*, 2008 ▶) using an iridium derivative. In both cases the models were built directly into the experimentally phased map.

## 3. Results   

### 3.1. Program overview   

**Figure 1:**
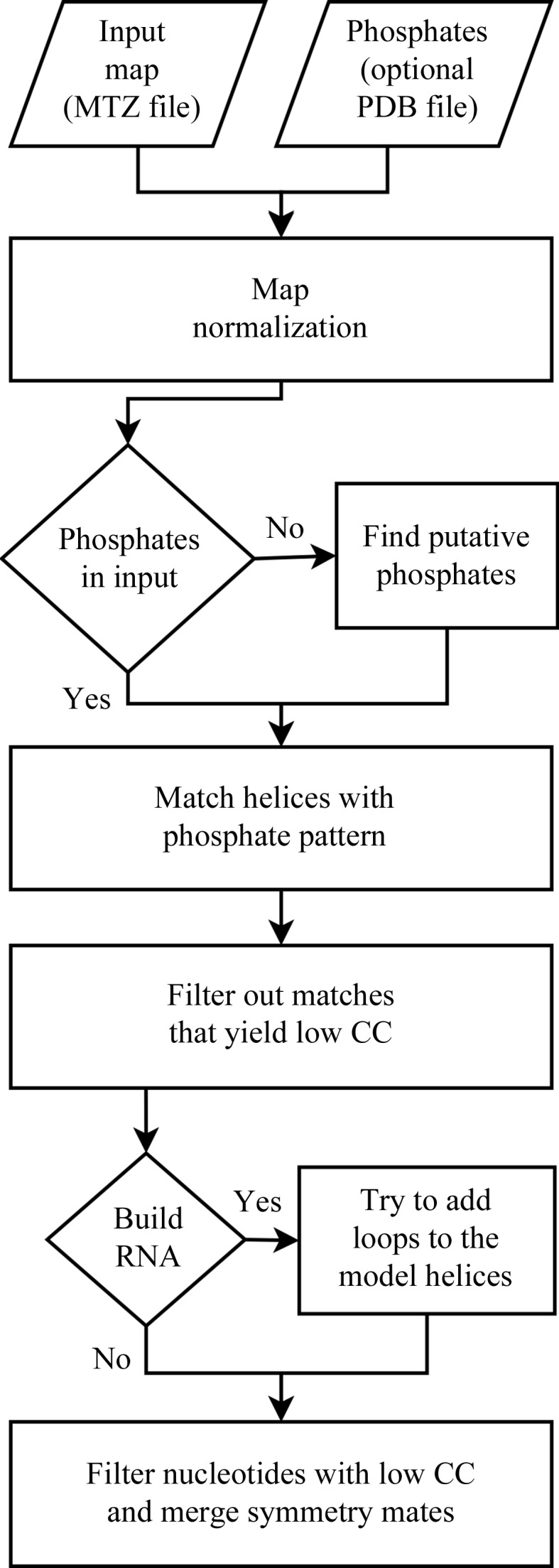
*Brickworx* algorithm flowchart.

*Brickworx* requires a (binary) MTZ file with structure-factor amplitudes and phases (Fig. 1 ▶). The program is able to predict the P-atom positions with the help of a support vector machine classifier and can also accept positions of P atoms that are specified by the user. In the latter case, the program can read P-atom positions from a user-defined file in PDB format. Since the quality of the input P-atom positions is crucial for the successful use of the program, the predicted pattern should be revised manually in difficult cases. The user must also specify whether the target structure is RNA or DNA. This is required for the determination of the correct double-helix geometry for building an initial model. Furthermore, if a target molecule is RNA, *Brickworx* will additionally try to build nonhelical recurrent motifs derived from the RNA Bricks database. On output *Brickworx* provides two files in PDB format: the predicted P-atom pattern (if applicable) and a model in a full-atom representation.

#### 3.1.1. Building nucleic acid models   

Each session of model building starts with the detection of putative phosphates in the map (Fig. 1 ▶). Next, the phosphate pattern is reduced to an asymmetric unit with a buffer to enhance the probability of finding matching triplets in the initial step of phosphate-based matching of the nucleic acid fragments. Later, a complete crystallographic environment of each atom is used.

#### 3.1.2. Building double helices (A-RNA and B-DNA)   

Firstly, the initial matches of the model double-helix P-atom and the target P-atom patterns are found (see §[Sec sec2]2 for details). This step yields a large fraction of false-positive solutions that are filtered out based on the quality of the fit to the electron density. The matches are scored with a sum of interpolated map values at the atom centres. The ten solutions with the highest score are further refined in real space with secondary-structure restraints. During refinement, two isosteric variants of each W–C base pair are tested to find the one that yields the best fit to the electron-density map. The isosteric base pairs display nearly the same C1′–C1′ distance and have their glycosidic bonds oriented in the same way, and can replace each other without significant changes in the phosphate-sugar backbone geometry (Leontis *et al.*, 2002 ▶). Finally, helices with a real-space correlation coefficient to the target map of 0.5 or above are selected. The procedure is iteratively repeated for double-stranded helix models of four and three base pairs.

#### 3.1.3. Building custom RNA loops   

If a target structure is RNA, the double-helical fragments matched in the first step are further expanded with recurrent RNA loop motifs. Firstly, all flanking W–C base pairs of a motif are superposed onto the terminal base pairs in the matched stems. Next, the initial match is fine-tuned based on the P-atom positions. In a similar way as the previous step, the ten best matches are further refined in real space with the secondary-structure restraints (including noncanonical base pairs defined with *ClaRNA*; Waleń *et al.*, 2014 ▶). All isosteric variants of the detected base pairs are tested to find the one that best fits the input electron density. Finally, a set of symmetry-unique nucleotides with a real-space correlation coefficient to the target map of above 0.6 is selected for output.

### 3.2. *Brickworx* phosphate-detection algorithm benchmarks   

The quality of the phosphate-group detection algorithm implemented in *Brickworx* was compared with the corresponding feature of *Knuspr* (Gruene & Sheldrick, 2011 ▶). For each map from the test set, P-atom patterns were detected using the two programs and were compared with the reference structure. Two parameters describing the prediction quality were estimated: precision, defined as the fraction of predicted P atoms that are correct (see §[Sec sec2]2 for details), and completeness, defined as the fraction of reference structure phosphates that were correctly predicted. For each pair of map parameters (resolution and mean phase error), the average value of the two prediction-quality parameters for a given reference-structure type (DNA, DNA with protein, RNA or RNA with protein) was estimated.

**Figure 2:**
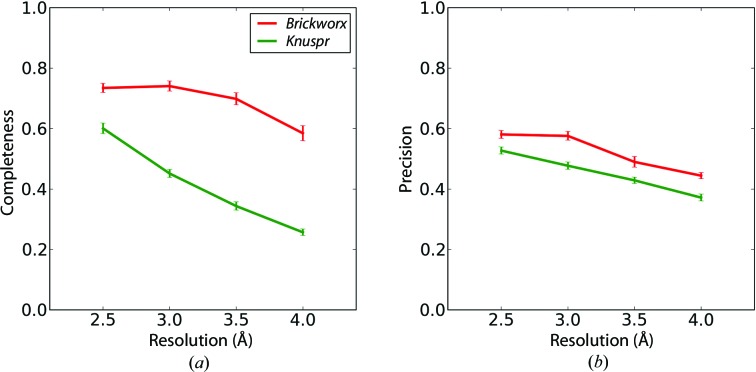
Completeness (*a*) and precision (*b*) of the phosphate-group detection algorithms implemented in *Knuspr* and *Brickworx* (red and green lines, respectively). The results presented in the figures are based on maps calculated for the RNA-only structures with a mean phase error and figure of merit of 35° and 0.75, respectively.

For all of the reference structure types, the *Brickworx* predictions have higher completeness than those from *Knuspr* (Fig. 2 ▶ and Supplementary Figs. S1–S12). The difference is larger for both low resolution and large mean phase error. In contrast, the precisions of the two prediction methods are comparable, regardless of the crystal structure composition and map quality. Furthermore, the prediction qualities of both methods are notably worse for structures that contain a protein component (see, for example, Supplementary Figs. S1 and S7).

### 3.3. *Brickworx* benchmarks for nucleic acid structures   

The quality of the polynucleotide model-building algorithm implemented in *Brickworx* was compared with the corresponding features of *ARP*/*wARP* (Hattne & Lamzin, 2008 ▶), *Nautilus* (Cowtan, 2012 ▶), *phenix.find_helices_strands* and *phenix.build_rna_helices* (RNA-containing structures only) (Terwilliger, 2010 ▶). Each of the maps from the test-set models was built using either of the methods and compared with the reference structure using the strict criteria defined in Gruene & Sheldrick (2011 ▶) or a relaxed scheme that does not test whether the base types in the model agree with the reference structure (see §[Sec sec2]2 for details). The introduction of the relaxed scheme was mainly dictated by the fact that *Nautilus* does not fit base types by design. The average precision and completeness of the predictions were estimated for each set of map parameters (resolution and mean phase error) and reference-structure type (DNA, DNA with protein, RNA and RNA with protein).

#### 3.3.1. Nucleic acid-only structures   

**Figure 3:**
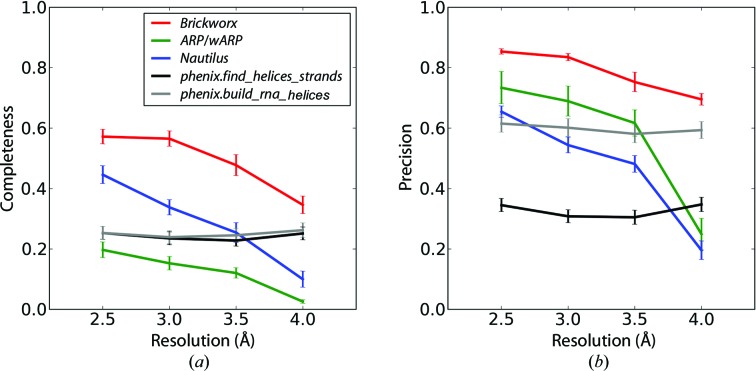
Completeness (*a*) and precision (*b*) of the model-building algorithms implemented in *Brickworx*, *ARP*/*wARP*, *Nautilus*, *phenix.find_helices_strands* and *phenix.build_rna_helices* (red, green, blue, black and grey lines, respectively). Only the P and C1′ atom position were evaluated in the output models. The results presented in the figures are based on maps calculated for the RNA-only structures with a mean phase error and figure of merit of 35° and 0.75, respectively.

For most of the RNA-only and DNA-only reference-structure types, *Brickworx* yields higher completeness and precision than the other methods in most cases (Fig. 3 ▶ and Supplementary Figs. S13–S18 and S19–S24). There are two exceptions. For maps calculated at resolutions of 3.0 Å and above with a relatively low mean phase error of 18°, the *ARP*/*wARP* DNA/RNA model-building module yields better precision than any of the methods tested. Furthermore, *phenix.build_rna_helices* tends to be weakly sensitive to large phase errors. This method readily outperforms other tools in terms of completeness for maps with a mean phase error of 56°. However, it should be noted that *phenix.build_rna_helices* exhibits lower precision than *Brickworx*.

#### 3.3.2. Protein–nucleic acid complexes   

**Figure 4:**
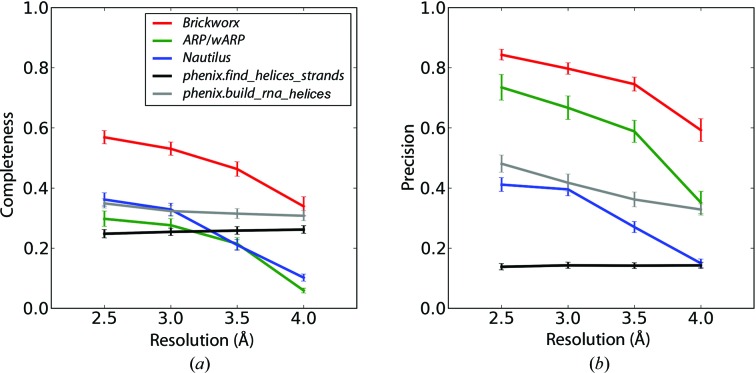
Completeness (*a*) and precision (*b*) of the model-building algorithms implemented in *Brickworx*, *ARP*/*wARP*, *Nautilus*, *phenix.find_helices_strands* and *phenix.build_rna_helices* (red, green, blue, black and grey lines, respectively). Only the P and C1′ atom position were evaluated in the output models. The results presented in the figures are based on maps calculated for the protein–RNA complexes with a mean phase error and figure of merit of 35° and 0.75, respectively.

**Figure 5:**
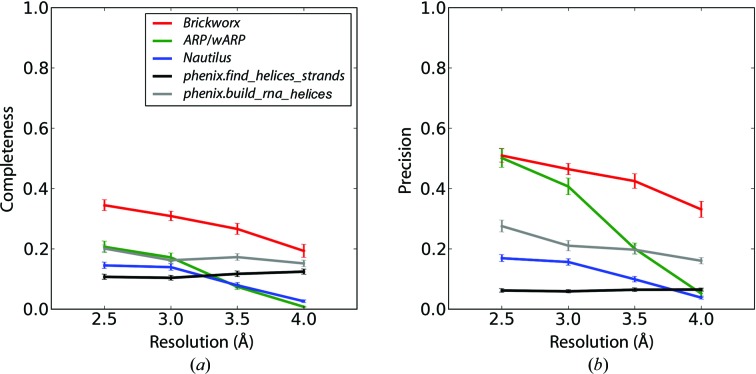
Completeness (*a*) and precision (*b*) of the model-building algorithms implemented in *Brickworx*, *ARP*/*wARP*, *Nautilus*, *phenix.find_helices_strands* and *phenix.build_rna_helices* (red, green, blue, black and grey lines, respectively). The model quality was evaluated based on a strict criterion (including base type and position). The results presented in the figures are based on maps calculated for the protein–RNA complexes with a mean phase error and figure of merit of 35° and 0.75, respectively.

For the RNA–protein and DNA–protein complexes, *Brickworx* performs better than *Nautilus* and *phenix.find_helices_strands* for all data sets tested. It yields better completeness than *ARP*/*wARP* if the mean phase error exceeds 18° and at resolutions below 3.0 Å (Fig. 4 ▶ and Supplementary Figs. S25–S36). *ARP*/*wARP* models, however, have better precision for data sets calculated at high and medium resolutions (above 3.0 Å) with a mean phase error of 18° (Fig. 5 ▶). Finally, for RNA–protein complex structures, models built using *phenix.build_rna_helices* cover the largest fraction of reference structures for maps with a mean phase error of 58°. On the other hand, models built with *Brickworx* exhibit significantly higher precision.

### 3.4. Tests with experimentally phased maps   

In this work, we sought to present detailed benchmarks for *Brickworx* that will reduce the subjectivity in the choice of test cases. Therefore, the simulated data sets covered a wide spectrum of structure types, resolutions and phase-information qualities. This approach provided data on the average performance of the tested methods, which is rarely reported in other studies. Detailed benchmark results for two experimental maps are also presented.

For the tests with experimental maps, we used a final version of *Brickworx* with a complete set of recurrent RNA motifs. A compilation of benchmark results are presented in Supplementary Tables S1 and S2. Both models were built using the webserver version of *Brickworx*. The computations took 20 and 7 min to analyze the group II intron and the lysine riboswitch maps, respectively.

#### 3.4.1. Group II intron   

**Figure 6:**
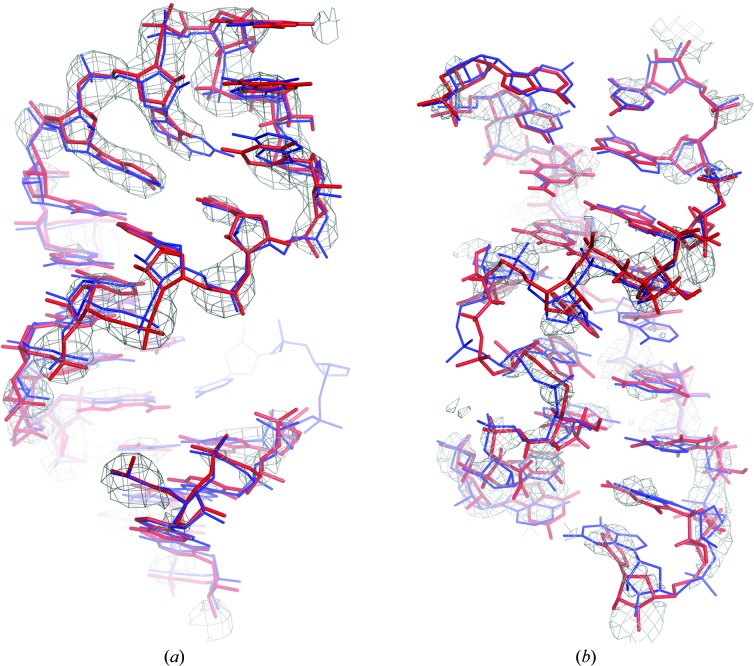
Comparison of published coordinates (blue) and crystal structure models built using *Brickworx* (red). (*a*) The GCGA tetraloop from the group II intron IC subdomain (Toor *et al.*, 2008 ▶). The model was fitted into the experimentally phased map (3.1 Å resolution) shown contoured at 3.0σ. The model is composed of a model stem generated with 3*DNA* (Lu & Olson, 2008 ▶) and a tetraloop extracted from SSU (PDB entry 1n33). (*b*) The sarcin–ricin motif from the lysine riboswitch (Garst *et al.*, 2008 ▶) fitted into an experimentally phased map (2.8 Å resolution) shown contoured at 1.8σ. The matched motif was extracted from the LSU structure (PDB entry 3j62) and further refined in real space with secondary-structure restraints defined using *ClaRNA* (Waleń *et al.*, 2014 ▶).

A crucial step in the *Brickworx* algorithm is detection of the phosphate-group positions in the unit cell. These are later used to guide the building of double-helical fragments into a map. In the group II intron map, *Brickworx* detected 234 out of 388 P-atom positions correctly (see §[Sec sec2.5]2.5 for details) with a precision as high as 75%. This resulted in a good-quality model that covered a large fraction of the published coordinates (Fig. 6 ▶ *a*). The model consists of 233 nucleotides, of which 179 (77%) have the correct nucleotide position, covering 46% of the reference structure. Moreover, 119 nucleotides have the correctly predicted base type (see §[Sec sec2.5]2.5 for a detailed description of the model-assessment procedure).

Compared with other methods, *ARP*/*wARP* was able to build a model that covered the largest fraction of the reference structure. From 234 nucleotides, 84 (36%) have the correct nucleotide position and 25 (11%) have the correct base type. In contrast, *phenix.build_rna_helices* returned a model that had the highest fraction of correctly built nucleotides: 72 nucleotides out of 114 (63%) were correctly placed and 45 (40%) have also a correctly predicted base type. Detailed benchmark results are available in Supplementary Table S1.

It must be emphasized that the presented model was built using a final webserver version of *Brickworx* with a complete set of RNA motifs. As a result, many recurrent motifs in the resulting model were originally extracted from other models of group II introns (*e.g.* PDB entry 4faw), which clearly biased the results. On the other hand, *Brickworx* was able to build a more complete model than any other tested method with an A-RNA model alone (Supplementary Table S1).

#### 3.4.2. Lysine riboswitch   

The quality of the lysine ribo­switch experimental map is evidently lower than that available for the group II intron. This is reflected in the results of the phosphate-detection procedure. *Brickworx* was able to correctly detect 61 out of 161 phosphate groups in the reference structure with a relatively low precision of 22%. As a result, the final model consists of 51 nucleotides, 41 of which (80%) have the correct position and 13 of which (25%) have the correctly predicted base type. These cover 25 and 8% of the reference structure, respectively.

Results obtained using other methods confirm the difficulty of this test case. The best model was obtained from *phenix.build_rna_helices* and consisted of 64 nucleotides in total: 37 (61%) nucleotides have the correct position and 18 (28%) have the correct base type. These cover 23 and 11% of the reference structure, respectively. Detailed benchmark results are available in Supplementary Table S2.

In contrast to the previous example, the lysine riboswitch model built using *Brickworx* does not contain any motifs from related structures. The largest nonhelical motif found by the program, the sarcin–ricin loop (Fig. 6 ▶), was originally extracted from the LSU structure (PDB entry 3j62).

## 4. Discussion   

### 4.1. The phosphate-detection step is crucial for the model-building procedure   

For *Brickworx*, the detection of phosphate-group positions is crucial for building fragments into the electron-density map. Although the program can handle a relatively large numbers of false positives, the number of correct predictions should be large. For this reason, we implemented our own procedure for the identification of phosphate groups in the electron-density maps based on a support vector machine classifier. The classifier was trained to provide high completeness of the predictions at the expense of precision. According to the benchmarks presented in §[Sec sec3]3, the method correctly identifies over 80% of the reference-structure phosphate groups at resolutions as low as 4.0 Å when the figure-of-merit values are high (Fig. 2 ▶ and Supplementary Figs. S1–S12). For low-quality maps, however, it can still identify 30% of the reference-structure phosphate groups correctly. This is sufficient to build over 10% of the reference-structure nucleotides correctly in cases when other model-building methods return no results.

The *Brickworx* feature of building crystal structure models of nucleic acids starting from the phosphate-group positions may be particularly useful in the case of the single-wavelength anomalous dispersion approach based on the anomalous signal of P atoms (P-SAD). This phasing technique yields the positions of P atoms in a unit cell. *Brickworx* could be used in cases in which only a fraction of the P atoms were found and the corresponding electron-density map is difficult to interpret.

### 4.2. *Brickworx* requires the presence of double-stranded RNA/DNA helices in a crystal   

The general problem of approximately matching two sets of points in space is computationally expensive. In our approach, reduction of the computation time was possible owing to the use of effective data structures (KD-trees) and algorithms (graph matching). Even so, it may be used for finding initial matching of just a few models in reasonable time. The overall number of unique loop fragments (exceeding 2000) is prohibitively large for this purpose. Therefore, in *Brickworx*, RNA double helices are first fitted into the electron-density maps and later used to find the correct positions of the loop motifs.

Regular double-stranded helices are relatively common in nucleic acid structures. Using the 3*DN*A suite, we have found that 84% of protein–RNA complexes, 95% of RNA-only structures, 91% of DNA-only structures and 92% of protein–DNA complexes deposited in the PDB (as of 1 August 2014) contain at least a single double helix in A or B conformation.

### 4.3. *Brickworx* is capable of building models of nucleic acids complexed to proteins   

The presence of a protein component in a crystal readily affects the precision of the phosphate-detection algorithm implemented in *Brickworx* (Fig. 2 ▶ and Fig. S11). However, the program can handle a relatively large fraction of false phosphate-group predictions and therefore the presence of a protein in the structure does not affect the success rate of the nucleic acid model-building procedure. In this context, the performance of *Brickworx* is comparable with the other two benchmarked programs. It must be mentioned, however, that regardless of whether a protein component was present in the crystal or not, the completeness and precision of *Brickworx* models are comparable at medium and better at high resolution compared with the other two benchmarked programs.

### 4.4. *Brickworx* builds models at low resolution   

*Brickworx* fits complete fragments of RNA and DNA tertiary structure that are composed of six or more nucleotides into electron-density maps. The fragments are further refined using secondary-structure restraints, which enables an optimal fit of the set of nucleotides to be found even at very low resolution, when a fraction of the residues in a motif are very poorly resolved. An analogous approach implemented in *phenix.build_rna_helices* yields correct models when the phase quality is very low, regardless the data-set resolution. This method, however, currently works only with RNA structures and can build exclusively double-stranded helices. In contrast, other tested programs such as *Nautilus* and *ARP*/*wARP* rely on finding local features of the electron density (phosphates and bases or sugar rings) to merge them into a continuous chain. This approach works best when both moieties can be readily resolved in the map. On the other hand, *Nautilus* and *ARP*/*wARP* can build purely single-stranded structures, which is currently not possible with our approach.

## 5. Conclusions   

*Brickworx* can build models of nucleic acid crystal structures by fitting recurrent structural motifs into the electron-density maps. The quality of the models built by the program and by *ARP*/*wARP*, *Nautilus*, *phenix.find_helices_strands* or *phenix.build_rna_helices* was compared using over 2000 electron-density maps calculated for a set of 193 high- and medium-resolution crystal structure models. According to the test results, *Brickworx* models are comparable in both completeness and quality to those from *ARP*/*wARP* at medium resolution, when nucleic acid features are readily visible in the maps. However, at low resolution *Brickworx* can build models that cover larger fractions of the target structure with a larger fraction of correctly built nucleotides. The program can also build models for data sets with a mean phase error above 50°, which is possible with *phenix.build_rna_helices* for RNA structures but rarely with other methods. *Brickworx* models also have a large fraction of nucleotides that are built with a proper base type, even though the target-sequence information is not taken into account.

The results presented in this work suggest that the approach implemented in *Brickworx* provides a suitable basis for future development. In particular, we plan to use sequence information to search for the conformations of single-stranded fragments connecting the motifs fitted by the program. In the current version, however, *Brickworx* may already provide valuable, high-quality starting models for both manual and automated model-building methods.

## Supplementary Material

Supporting Information.. DOI: 10.1107/S1399004715000383/wa5082sup1.pdf

## References

[bb1] Adams, P. D. *et al.* (2010). *Acta Cryst.* D**66**, 213–221.

[bb2] Bernstein, F. C., Koetzle, T. F., Williams, G. J., Meyer, E. F., Brice, M. D., Rodgers, J. R., Kennard, O., Shimanouchi, T. & Tasumi, M. (1977). *Eur. J. Biochem.* **80**, 319–324. 10.1111/j.1432-1033.1977.tb11885.x 923582

[bb3] Cech, T. R. & Steitz, J. (2014). *Cell*, **157**, 77–94. 10.1016/j.cell.2014.03.008 24679528

[bb4] Chojnowski, G., Walen, T. & Bujnicki, J. M. (2014). *Nucleic Acids Res.* **42**, D123–D131. 10.1093/nar/gkt1084 PMC3965019 24220091

[bb5] Cowtan, K. (2012). *CCP4 Newsl. Protein Crystallogr.* **48**, contribution 6.

[bb6] Dezső, B., Jüttner, A. & Kovács, P. (2011). *Electron. Notes Theor. Comput. Sci.* **264**, 23–45.

[bb7] Doudna, J. (2000). *Nature Struct. Biol.* **7**, 954–956. 10.1038/80729 11103998

[bb8] Emsley, P., Lohkamp, B., Scott, W. G. & Cowtan, K. (2010). *Acta Cryst.* D**66**, 486–501. 10.1107/S0907444910007493 PMC2852313 20383002

[bb9] Garst, A. D., Héroux, A., Rambo, R. P. & Batey, R. T. (2008). *J. Biol. Chem.* **283**, 22347–22351. 10.1074/jbc.C800120200 PMC2504901 18593706

[bb10] Grosse-Kunstleve, R. W., Sauter, N. K., Moriarty, N. W. & Adams, P. D. (2002). *J. Appl. Cryst.* **35**, 126–136.

[bb11] Gruene, T. & Sheldrick, G. M. (2011). *Acta Cryst.* A**67**, 1–8. 10.1107/S0108767310039140 PMC3006036 21173468

[bb12] Hattne, J. & Lamzin, V. S. (2008). *Acta Cryst.* D**64**, 834–842. 10.1107/S0907444908014327 18645232

[bb13] Joosten, R. P., Joosten, K., Murshudov, G. N. & Perrakis, A. (2012). *Acta Cryst.* D**68**, 484–496. 10.1107/S0907444911054515 PMC3322608 22505269

[bb14] Ke, A. & Doudna, J. (2004). *Methods*, **34**, 408–414. 10.1016/j.ymeth.2004.03.027 15325657

[bb15] Keating, K. S. & Pyle, A. M. (2010). *Proc. Natl Acad. Sci. USA*, **107**, 8177–8182. 10.1073/pnas.0911888107 PMC2889552 20404211

[bb16] Kleywegt, G. J., Harris, M. R., Zou, J., Taylor, T. C., Wählby, A. & Jones, T. A. (2004). *Acta Cryst.* D**60**, 2240–2249. 10.1107/S0907444904013253 15572777

[bb17] Leontis, N. B., Stombaugh, J. & Westhof, E. (2002). *Nucleic Acids Res.* **30**, 3497–3531. 10.1093/nar/gkf481 PMC134247 12177293

[bb18] Leontis, N. B. & Zirbel, C. L. (2012). *RNA 3D Structure Analysis and Prediction*, edited by N. Leontis & E. Westhof, pp. 281–298. Berlin, Heidelberg: Springer.

[bb19] Lu, X.-J. & Olson, W. K. (2008). *Nature Protoc.* **3**, 1213–1227. 10.1038/nprot.2008.104 PMC3065354 18600227

[bb20] Terwilliger, T. C. (2010). *Acta Cryst.* D**66**, 268–275. 10.1107/S0907444910000314 PMC2827347 20179338

[bb21] Terwilliger, T. C., Grosse-Kunstleve, R. W., Afonine, P. V., Moriarty, N. W., Zwart, P. H., Hung, L.-W., Read, R. J. & Adams, P. D. (2008). *Acta Cryst.* D**64**, 61–69. 10.1107/S090744490705024X PMC2394820 18094468

[bb22] Toor, N., Keating, K. S., Taylor, S. D. & Pyle, A. M. (2008). *Science*, **320**, 77–82. 10.1126/science.1153803 PMC4406475 18388288

[bb23] Waleń, T., Chojnowski, G., Gierski, P. & Bujnicki, J. M. (2014). *Nucleic Acids Res.* **42**, e151. 10.1093/nar/gku765 PMC4231730 25159614

[bb24] Winn, M. D. *et al.* (2011). *Acta Cryst.* D**67**, 235–242.

[bb25] Yamashita, K., Zhou, Y., Tanaka, I. & Yao, M. (2013). *Acta Cryst.* D**69**, 1171–1179. 10.1107/S0907444913007191 23695261

