# Supplementary material for: Brickworx builds recurrent RNA and DNA structural motifs into medium- and low-resolution electron-density maps
Source: Acta Crystallogr D Biol Crystallogr. 2015 Feb 26;71(Pt 3):697–705. doi: 10.1107/S1399004715000383 (PMC4356372; doi:10.1107/S1399004715000383)
Supplement: Supplementary file 1 [file d-71-00697-sup1.pdf]

BIOLOGICAL
CRYSTALLOGRAPHY

Volume 71 (2015)

Supporting information for article:

Brickworx builds recurrent RNA and DNA structural motifs into medium- and low-resolution electron-density maps

Grzegorz Chojnowski, Tomasz Waleń, Pawel Piątkowski, Wojciech Potrzebowski and Janusz M. Bujnicki

Brickworx builds recurrent RNA/DNA structural motifs into medium and low-resolution electron density maps
Supplementary figures and data

Grzegorz Chojnowski, Tomasz Walen, Pawel Piatkowski, Wojciech Potrzebowski, and Janusz M. Bujnicki

December 4, 2014

Contents

1 Benchmarks of the phosphate group detection algorithms implemented in Knuspr and Brickworx **ii**

- 1.1 DNA-only structures . . . ii
- 1.2 RNA-only structures . . . iv
- 1.3 protein-DNA complexes . . . v
- 1.4 protein-RNA complexes . . . vii

2 Benchmarks of the model building algorithms implemented in Brickworx, ARP/wARP, and Nautilus **ix**

- 2.1 DNA-only structures (backbone position evaluated) . . . ix
- 2.2 DNA-only structures (base type and postion evaluated) . . . xi
- 2.3 RNA-only structures (backbone position evaluated) . . . xiii
- 2.4 RNA-only structures (base type and postion evaluated) . . . xv
- 2.5 protein-DNA complexes (backbone position evaluated) . . . xvii
- 2.6 protein-DNA complexes (base type and postion evaluated) . . . xix
- 2.7 protein-RNA complexes (backbone position evaluated) . . . xxi
- 2.8 protein-RNA complexes (base type and postion evaluated) . . . xxiii

3 Brickworx computation-time benchmarks **xxv**

4 Matching algorithm computational complexity estimates **xxvi**

5 Benchmarks with experimantal electron-denity maps **xxviii**

6 Support Vector Machine classifier training set structures **xxix**

7 Reference structures used for benchmarks **xxix**

1 Benchmarks of the phosphate group detection algorithms implemented in Knuspr and Brickworx

1.1 DNA-only structures

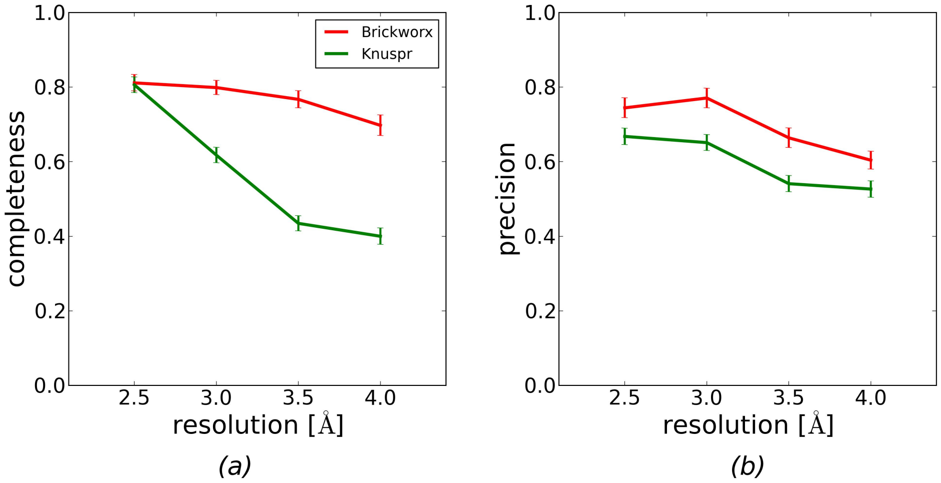

Figure S1: Completeness (a) and precision (b) of the phosphate group detection algorithms implemented in Knuspr and Brickworx (red, and green lines respectively). The results presented on the figures are based on maps calculated for the DNA-only structures with mean phase error and figure-of-merit of $\langle|\Delta\phi|\rangle = 18^\circ$ and $\langle m\rangle = 0.92$ respectively.

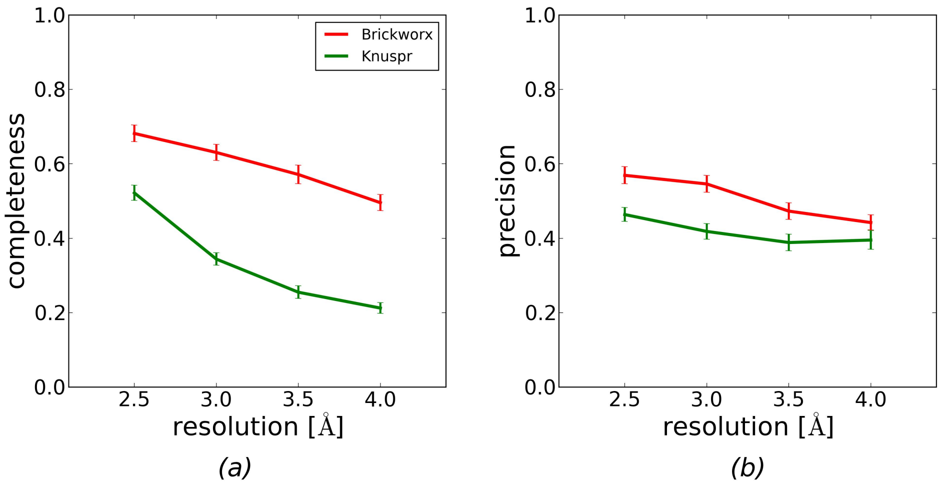

Figure S2: Completeness (a) and precision (b) of the phosphate group detection algorithms implemented in Knuspr and Brickworx (red, and green lines respectively). The results presented on the figures are based on maps calculated for the DNA-only structures with mean phase error and figure-of-merit of $\langle|\Delta\phi|\rangle = 35°$ and $\langle m \rangle = 0.75$ respectively.

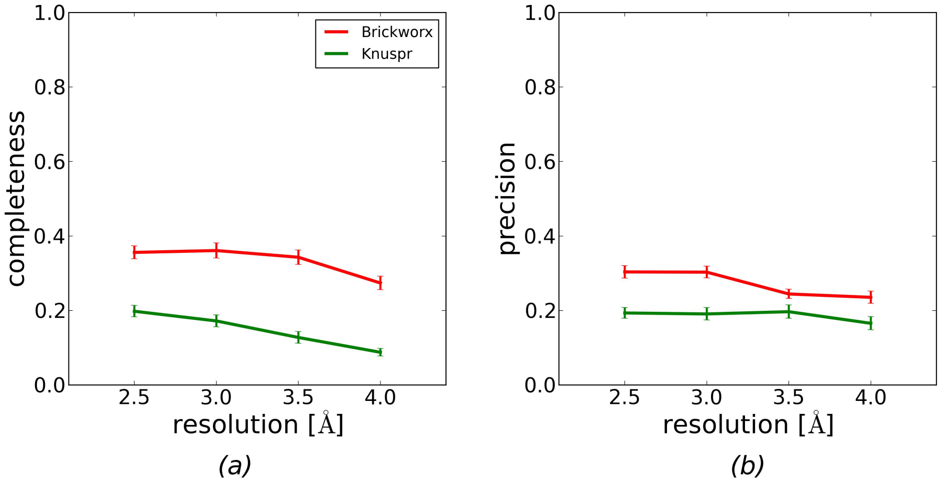

Figure S3: Completeness (a) and precision (b) of the phosphate group detection algorithms implemented in Knuspr and Brickworx (red, and green lines respectively). The results presented on the figures are based on maps calculated for the DNA-only structures with mean phase error and figure-of-merit of $\langle|\Delta\phi|\rangle = 54°$ and $\langle m \rangle = 0.50$ respectively.

1.2 RNA-only structures

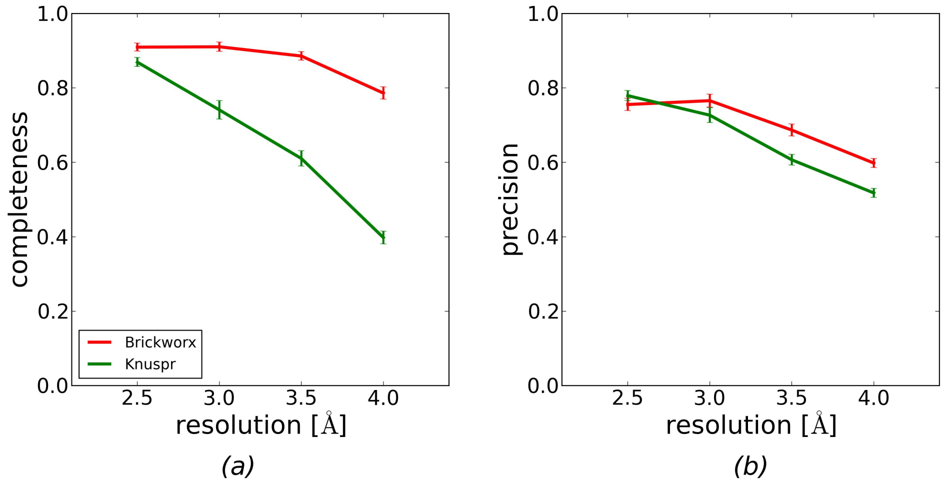

Figure S4: Completeness (a) and precision (b) of the phosphate group detection algorithms implemented in Knuspr and Brickworx (red, and green lines respectively). The results presented on the figures are based on maps calculated for the RNA-only structures with mean phase error and figure-of-merit of $\langle|\Delta\phi|\rangle = 18°$ and $\langle m \rangle = 0.92$ respectively.

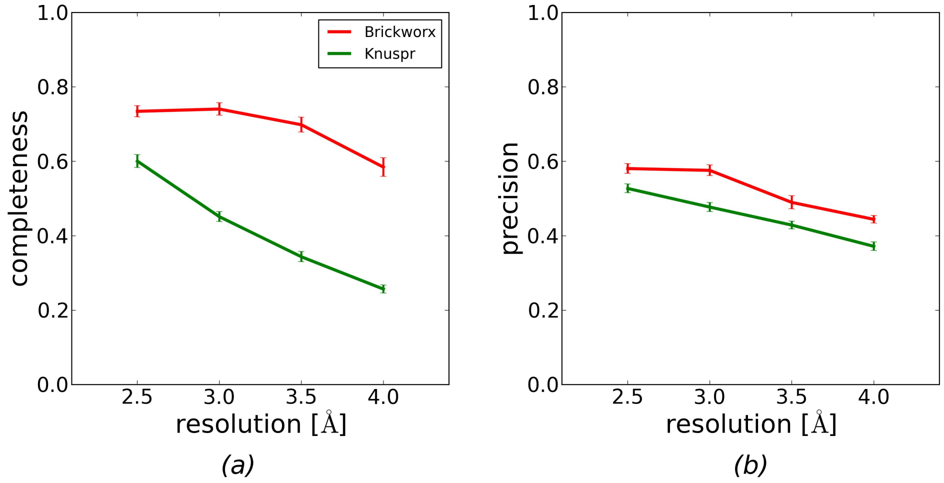

Figure S5: Completeness (a) and precision (b) of the phosphate group detection algorithms implemented in Knuspr and Brickworx (red, and green lines respectively). The results presented on the figures are based on maps calculated for the RNA-only structures with mean phase error and figure-of-merit of $\langle|\Delta\phi|\rangle = 35°$ and $\langle m \rangle = 0.75$ respectively.

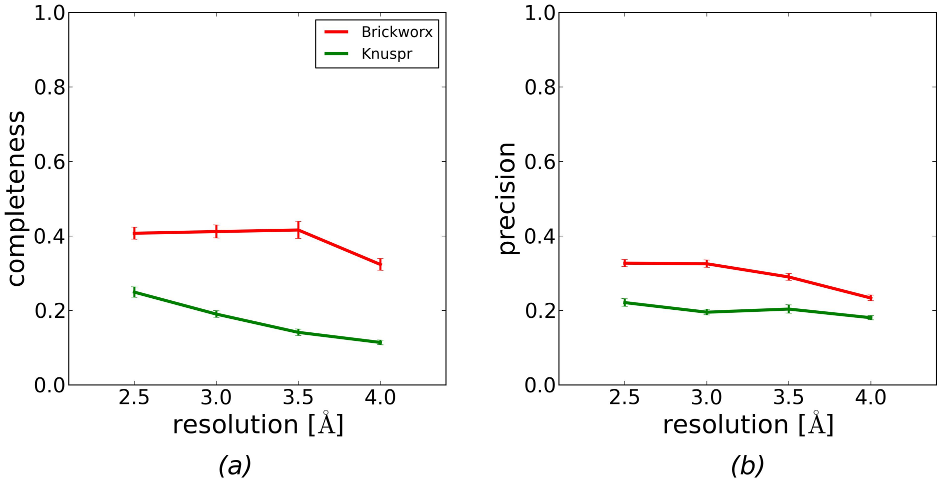

Figure S6: Completeness (a) and precision (b) of the phosphate group detection algorithms implemented in Knuspr and Brickworx (red, and green lines respectively). The results presented on the figures are based on maps calculated for the RNA-only structures with mean phase error and figure-of-merit of $\langle|\Delta\phi|\rangle = 54^{\circ}$ and $\langle m \rangle = 0.50$ respectively.

1.3 protein-DNA complexes

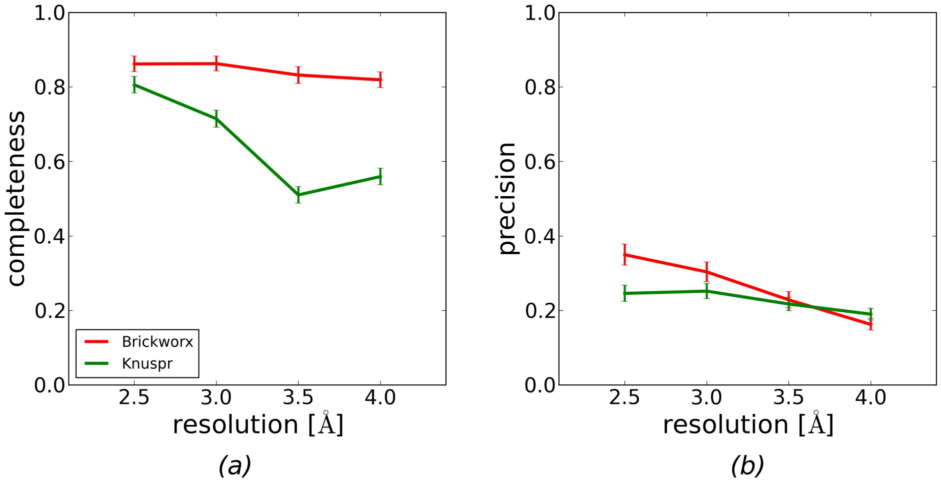

Figure S7: Completeness (a) and precision (b) of the phosphate group detection algorithms implemented in Knuspr and Brickworx (red, and green lines respectively). The results presented on the figures are based on maps calculated for the protein-DNA complexes with mean phase error and figure-of-merit of $\langle|\Delta\phi|\rangle = 18^{\circ}$ and $\langle m \rangle = 0.92$ respectively.

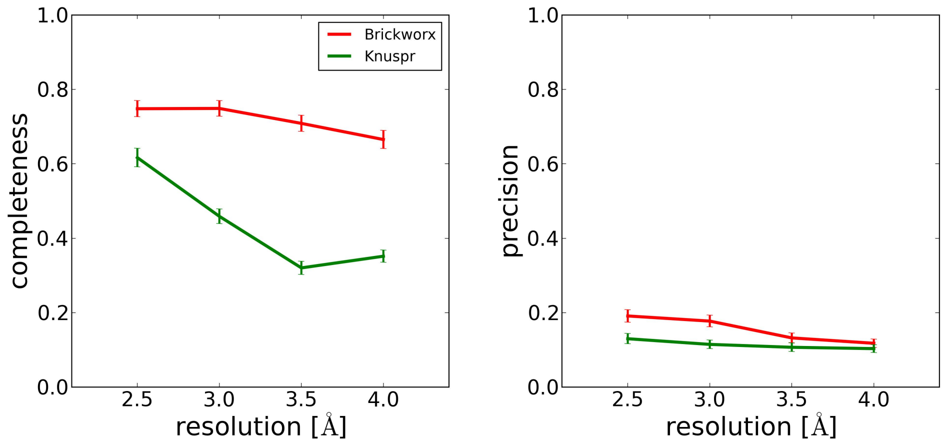

(a) *(b)*

Figure S8: Completeness (a) and precision (b) of the phosphate group detection algorithms implemented in Knuspr and Brickworx (red, and green lines respectively). The results presented on the figures are based on maps calculated for the protein-DNA complexes with mean phase error and figure-of-merit of $\langle|\Delta\phi|\rangle = 35°$ and $\langle m \rangle = 0.75$ respectively.

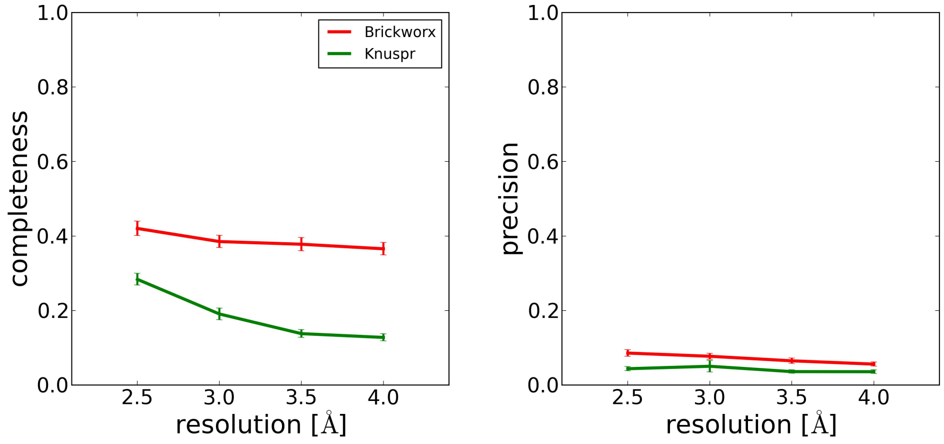

(a) *(b)*

Figure S9: Completeness (a) and precision (b) of the phosphate group detection algorithms implemented in Knuspr and Brickworx (red, and green lines respectively). The results presented on the figures are based on maps calculated for the protein-DNA complexes with mean phase error and figure-of-merit of $\langle|\Delta\phi|\rangle = 54°$ and $\langle m \rangle = 0.50$ respectively.

1.4 protein-RNA complexes

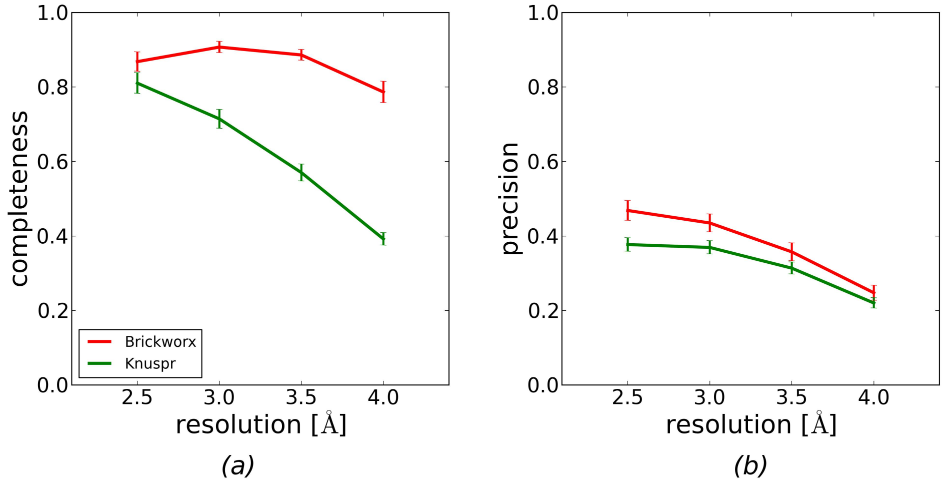

Figure S10: Completeness (a) and precision (b) of the phosphate group detection algorithms implemented in Knuspr and Brickworx (red, and green lines respectively). The results presented on the figures are based on maps calculated for the protein-RNA complexes with mean phase error and figure-of-merit of $\langle|\Delta\phi|\rangle = 18°$ and $\langle m\rangle = 0.92$ respectively.

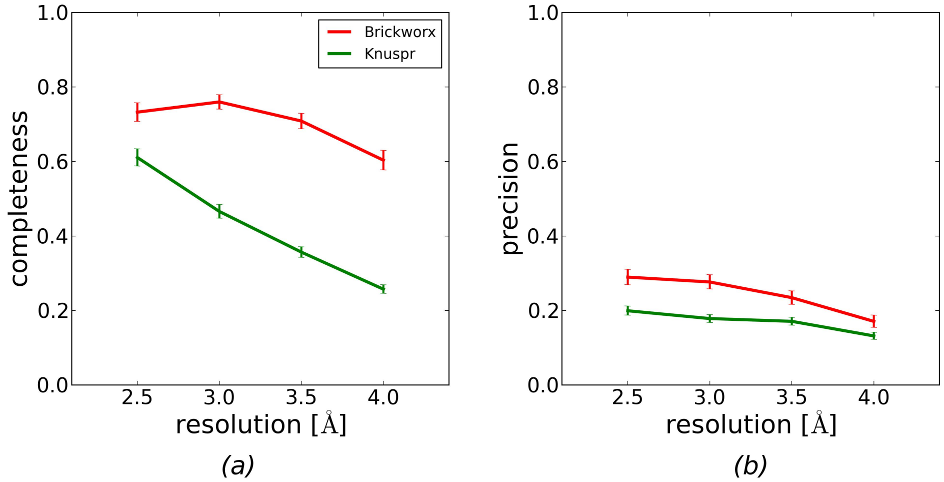

Figure S11: Completeness (a) and precision (b) of the phosphate group detection algorithms implemented in Knuspr and Brickworx (red, and green lines respectively). The results presented on the figures are based on maps calculated for the protein-RNA complexes with mean phase error and figure-of-merit of $\langle|\Delta\phi|\rangle = 35°$ and $\langle m\rangle = 0.75$ respectively.

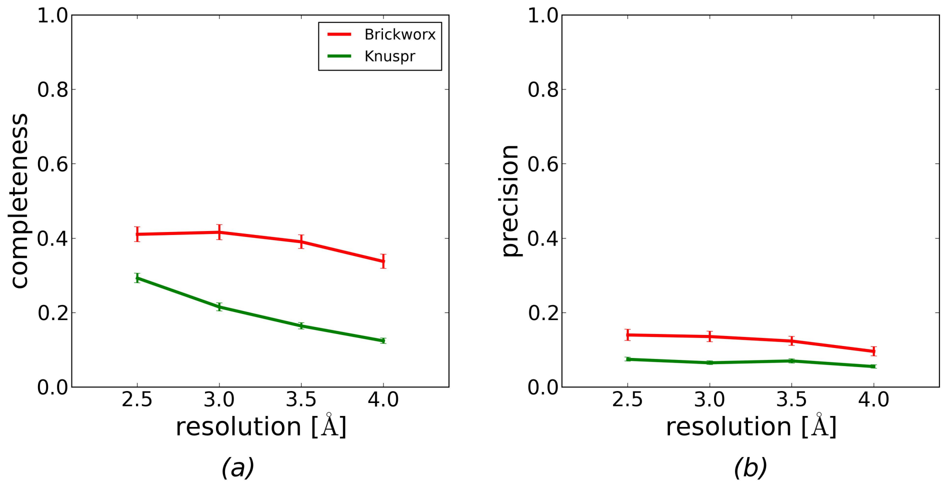

Figure S12: Completeness (a) and precision (b) of the phosphate group detection algorithms implemented in Knuspr and Brickworx (red, and green lines respectively). The results presented on the figures are based on maps calculated for the protein-RNA complexes with mean phase error and figure-of-merit of $\langle|\Delta\phi|\rangle = 54°$ and $\langle m\rangle = 0.50$ respectively.

2 Benchmarks of the model building algorithms implemented in Brickworx, ARP/wARP, and Nautilus

2.1 DNA-only structures (backbone position evaluated)

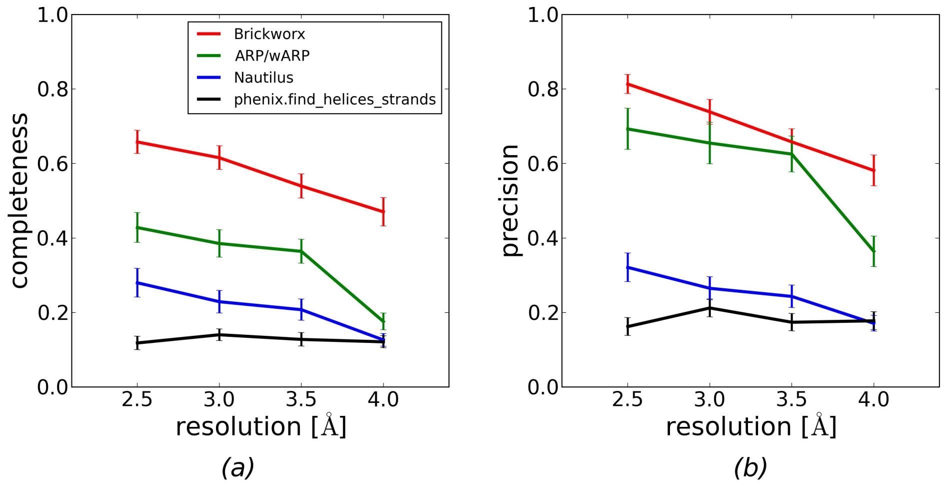

Figure S13: Completeness (a) and precision (b) of the model building algorithms implemented in Brickworx, ARP/wARP, and Nautilus (red, green, and blue lines frespectively). Only phosphorus and $C1'$ atom position were evaluated in the output models. The results presented on the figures are based on maps calculated for the DNA-only structures with mean phase error and figure-of-merit of $\langle|\Delta\phi|\rangle = 18^\circ$ and $\langle m \rangle = 0.92$ respectively.

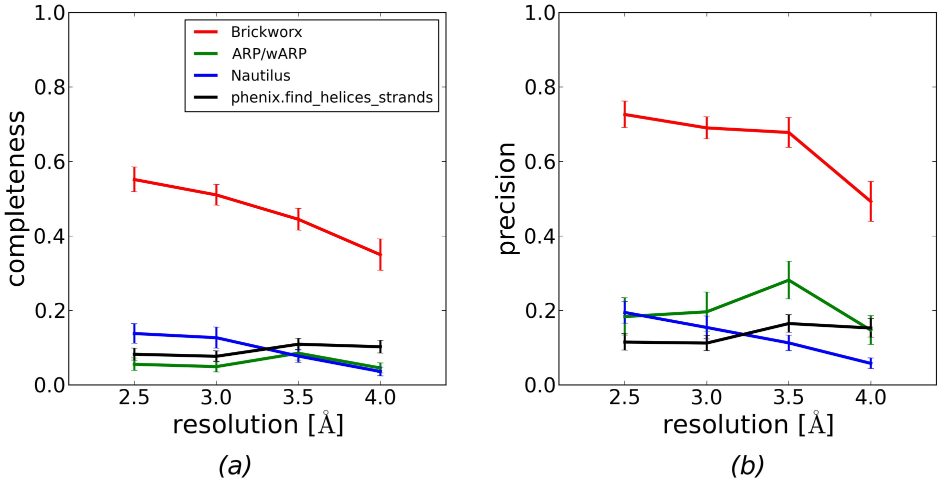

Figure S14: Completeness (a) and precision (b) of the model building algorithms implemented in Brickworx, ARP/wARP, and Nautilus (red, green, and blue lines frespectively). Only phosphorus and $C1'$ atom position were evaluated in the output models. The results presented on the figures are based on maps calculated for the DNA-only structures with mean phase error and figure-of-merit of $\langle|\Delta\phi|\rangle = 35°$ and $\langle m\rangle = 0.75$ respectively.

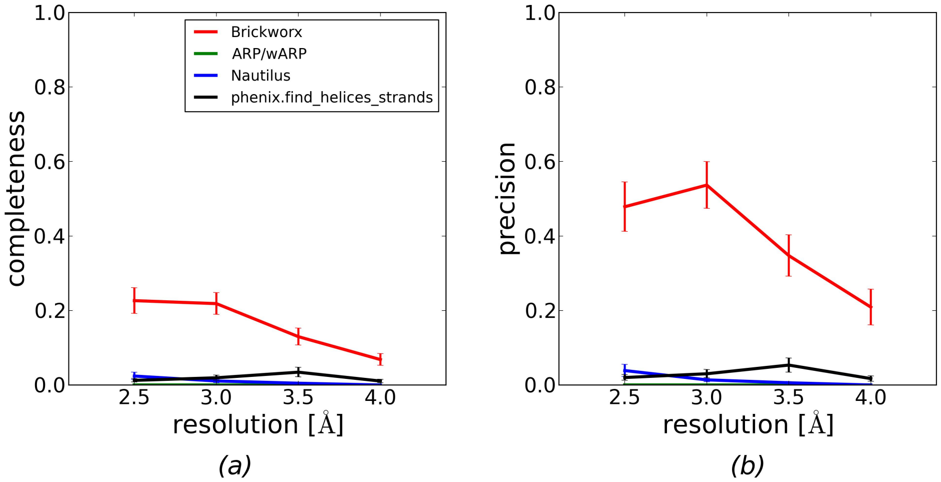

Figure S15: Completeness (a) and precision (b) of the model building algorithms implemented in Brickworx, ARP/wARP, and Nautilus (red, green, and blue lines frespectively). Only phosphorus and $C1'$ atom position were evaluated in the output models. The results presented on the figures are based on maps calculated for the DNA-only structures with mean phase error and figure-of-merit of $\langle|\Delta\phi|\rangle = 54°$ and $\langle m\rangle = 0.50$ respectively.

2.2 DNA-only structures (base type and postion evaluated)

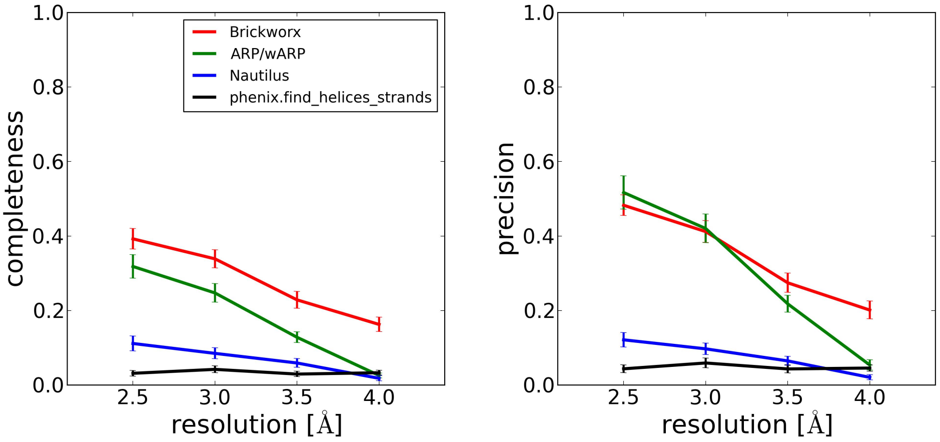

(a) (b)

Figure S16: Completeness (a) and precision (b) of the model building algorithms implemented in Brickworx, ARP/wARP, and Nautilus (red, green, and blue lines frespectively). The models quality was evaluated based on strict criterion (including base type and position). The results presented on the figures are based on maps calculated for the DNA-only structures with mean phase error and figure-of-merit of $\langle|\Delta\phi|\rangle = 18°$ and $\langle m\rangle = 0.92$ respectively.

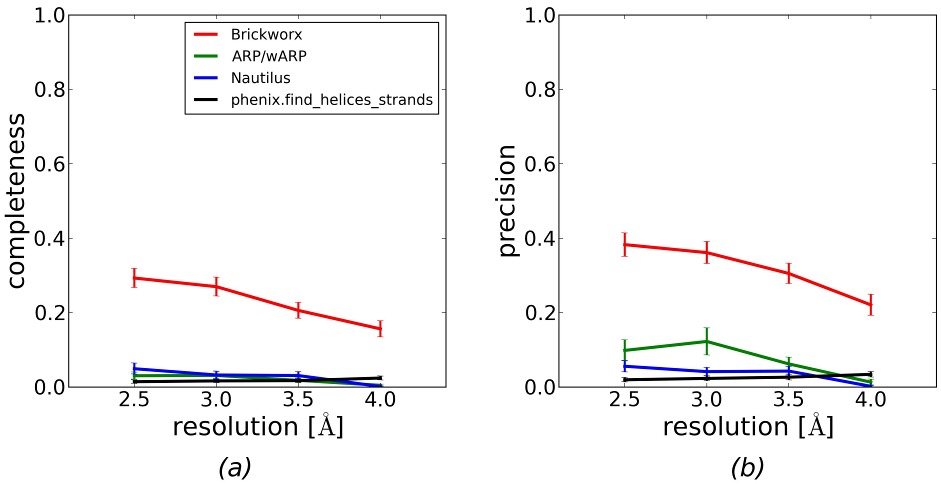

Figure S17: Completeness (a) and precision (b) of the model building algorithms implemented in Brickworx, ARP/wARP, and Nautilus (red, green, and blue lines frespectively). The models quality was evaluated based on strict criterion (including base type and position). The results presented on the figures are based on maps calculated for the DNA-only structures with mean phase error and figure-of-merit of $\langle|\Delta\phi|\rangle = 35^\circ$ and $\langle m\rangle = 0.75$ respectively.

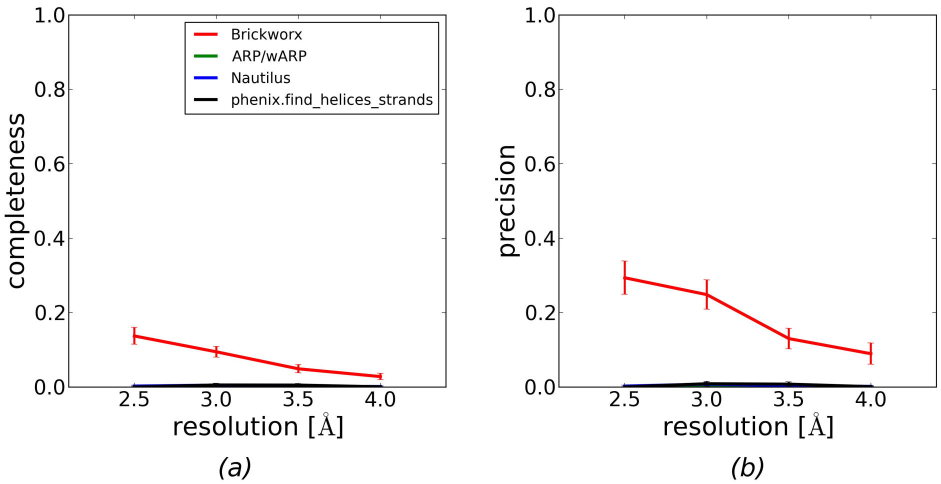

Figure S18: Completeness (a) and precision (b) of the model building algorithms implemented in Brickworx, ARP/wARP, and Nautilus (red, green, and blue lines frespectively). The models quality was evaluated based on strict criterion (including base type and position). The results presented on the figures are based on maps calculated for the DNA-only structures with mean phase error and figure-of-merit of $\langle|\Delta\phi|\rangle = 54^\circ$ and $\langle m\rangle = 0.50$ respectively.

2.3 RNA-only structures (backbone position evaluated)

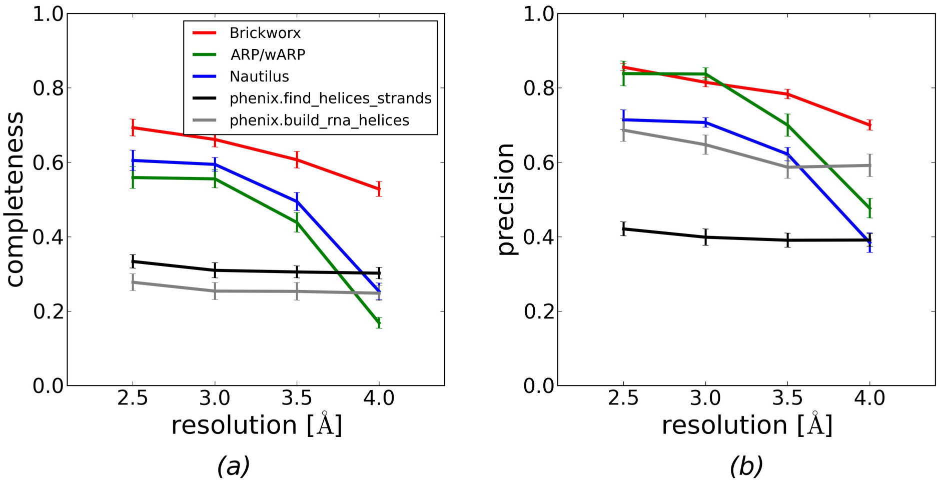

Figure S19: Completeness (a) and precision (b) of the model building algorithms implemented in Brickworx, ARP/wARP, and Nautilus (red, green, and blue lines frespectively). Only phosphorus and $C1'$ atom position were evaluated in the output models. The results presented on the figures are based on maps calculated for the RNA-only structures with mean phase error and figure-of-merit of $\langle|\Delta\phi|\rangle = 18°$ and $\langle m \rangle = 0.92$ respectively.

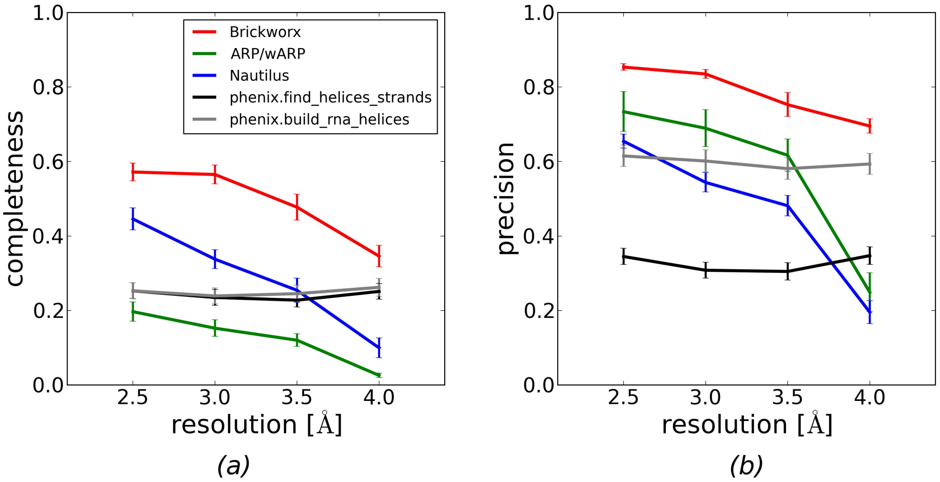

Figure S20: Completeness (a) and precision (b) of the model building algorithms implemented in Brickworx, ARP/wARP, and Nautilus (red, green, and blue lines frespectively). Only phosphorus and $C1'$ atom position were evaluated in the output models. The results presented on the figures are based on maps calculated for the RNA-only structures with mean phase error and figure-of-merit of $\langle|\Delta\phi|\rangle = 35°$ and $\langle m \rangle = 0.75$ respectively.

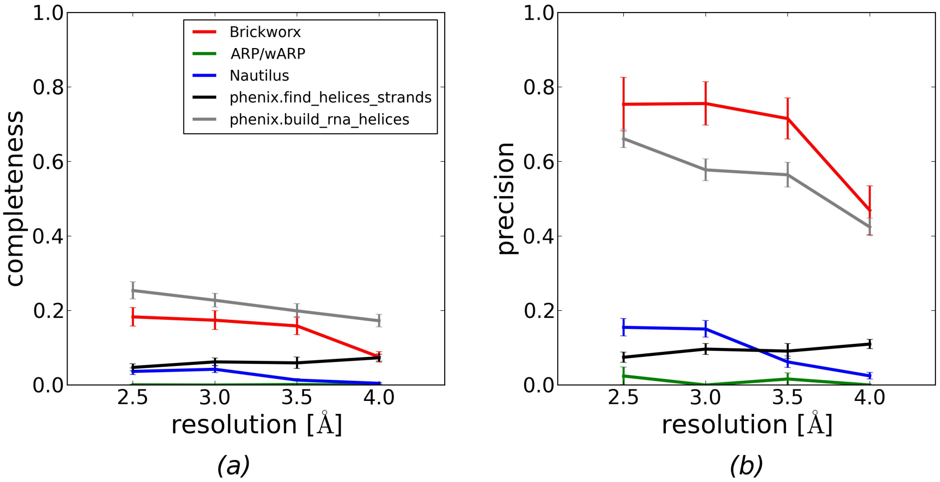

Figure S21: Completeness (a) and precision (b) of the model building algorithms implemented in Brickworx, ARP/wARP, and Nautilus (red, green, and blue lines frespectively). Only phosphorus and $C1'$ atom position were evaluated in the output models. The results presented on the figures are based on maps calculated for the RNA-only structures with mean phase error and figure-of-merit of $\langle|\Delta\phi|\rangle = 54°$ and $\langle m \rangle = 0.50$ respectively.

2.4 RNA-only structures (base type and postion evaluated)

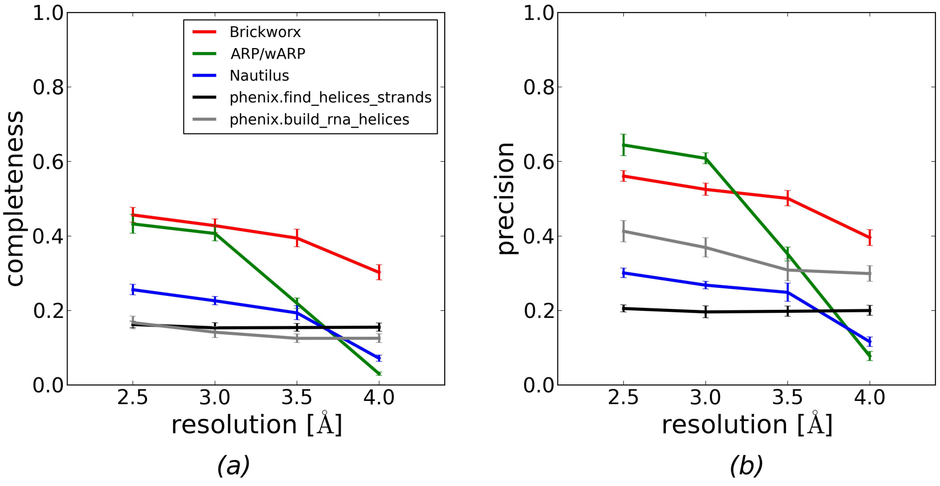

Figure S22: Completeness (a) and precision (b) of the model building algorithms implemented in Brickworx, ARP/wARP, and Nautilus (red, green, and blue lines frespectively). The models quality was evaluated based on strict criterion (including base type and position). The results presented on the figures are based on maps calculated for the RNA-only structures with mean phase error and figure-of-merit of $\langle|\Delta\phi|\rangle = 18^{\circ}$ and $\langle m \rangle = 0.92$ respectively.

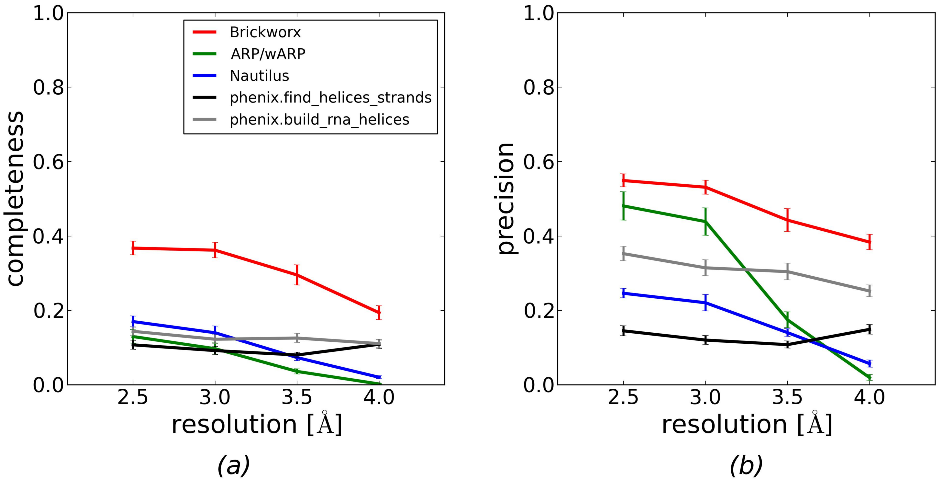

Figure S23: Completeness (a) and precision (b) of the model building algorithms implemented in Brickworx, ARP/wARP, and Nautilus (red, green, and blue lines frespectively). The models quality was evaluated based on strict criterion (including base type and position). The results presented on the figures are based on maps calculated for the RNA-only structures with mean phase error and figure-of-merit of $\langle|\Delta\phi|\rangle = 35^\circ$ and $\langle m \rangle = 0.75$ respectively.

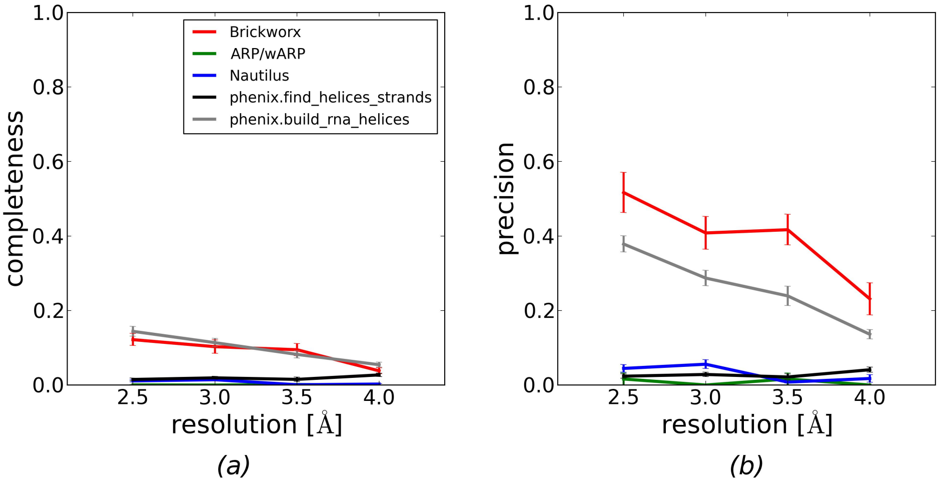

Figure S24: Completeness (a) and precision (b) of the model building algorithms implemented in Brickworx, ARP/wARP, and Nautilus (red, green, and blue lines frespectively). The models quality was evaluated based on strict criterion (including base type and position). The results presented on the figures are based on maps calculated for the RNA-only structures with mean phase error and figure-of-merit of $\langle|\Delta\phi|\rangle = 54^\circ$ and $\langle m \rangle = 0.50$ respectively.

2.5 protein-DNA complexes (backbone position evaluated)

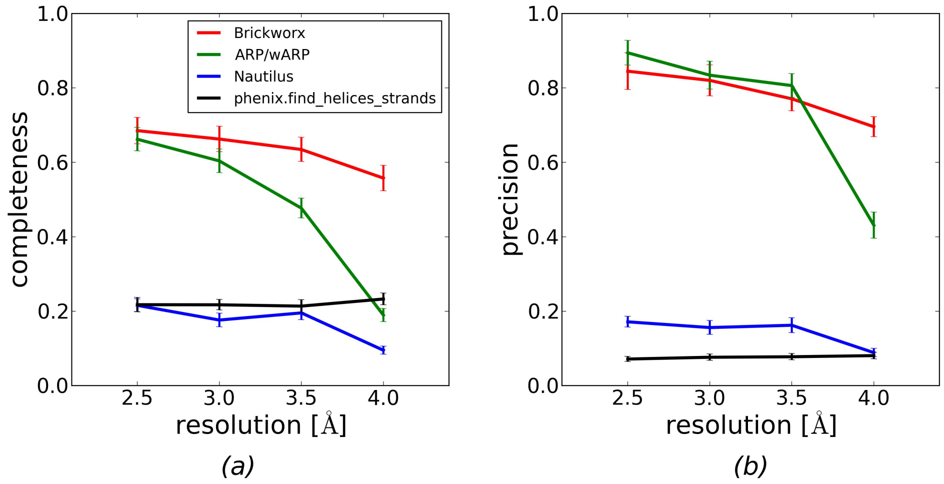

Figure S25: Completeness (a) and precision (b) of the model building algorithms implemented in Brickworx, ARP/wARP, and Nautilus (red, green, and blue lines frespectively). Only phosphorus and $C1'$ atom position were evaluated in the output models. The results presented on the figures are based on maps calculated for the protein-DNA complexes with mean phase error and figure-of-merit of $\langle|\Delta\phi|\rangle = 18°$ and $\langle m\rangle = 0.92$ respectively.

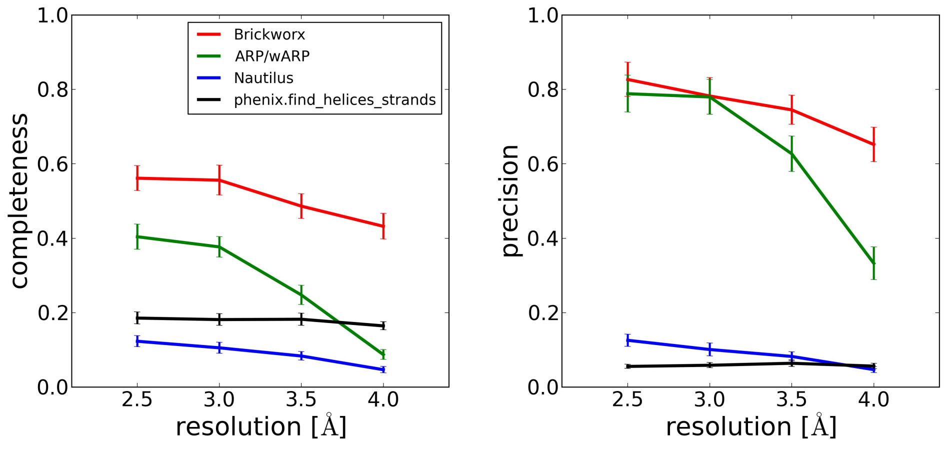

Figure S26: Completeness (a) and precision (b) of the model building algorithms implemented in Brickworx, ARP/wARP, and Nautilus (red, green, and blue lines frespectively). Only phosphorus and $C1'$ atom position were evaluated in the output models. The results presented on the figures are based on maps calculated for the protein-DNA complexes with mean phase error and figure-of-merit of $\langle|\Delta\phi|\rangle = 35^\circ$ and $\langle m \rangle = 0.75$ respectively.

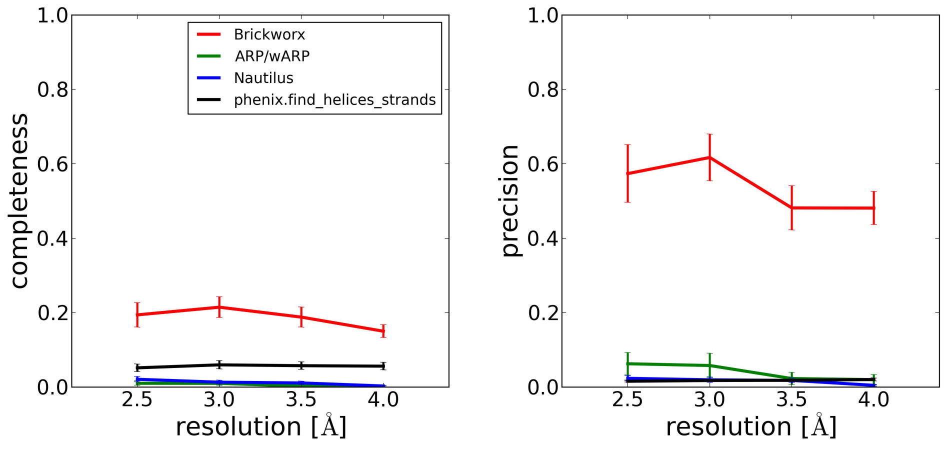

Figure S27: Completeness (a) and precision (b) of the model building algorithms implemented in Brickworx, ARP/wARP, and Nautilus (red, green, and blue lines frespectively). Only phosphorus and $C1'$ atom position were evaluated in the output models. The results presented on the figures are based on maps calculated for the protein-DNA complexes with mean phase error and figure-of-merit of $\langle|\Delta\phi|\rangle = 54^\circ$ and $\langle m \rangle = 0.50$ respectively.

2.6 protein-DNA complexes (base type and postion evaluated)

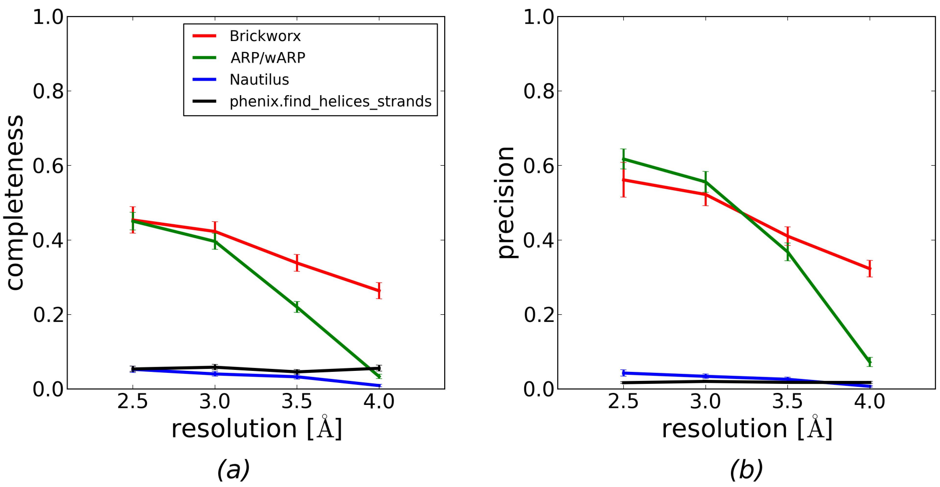

Figure S28: Completeness (a) and precision (b) of the model building algorithms implemented in Brickworx, ARP/wARP, and Nautilus (red, green, and blue lines frespectively). The models quality was evaluated based on strict criterion (including base type and position). The results presented on the figures are based on maps calculated for the protein-DNA complexes with mean phase error and figure-of-merit of $\langle|\Delta\phi|\rangle = 18°$ and $\langle m \rangle = 0.92$ respectively.

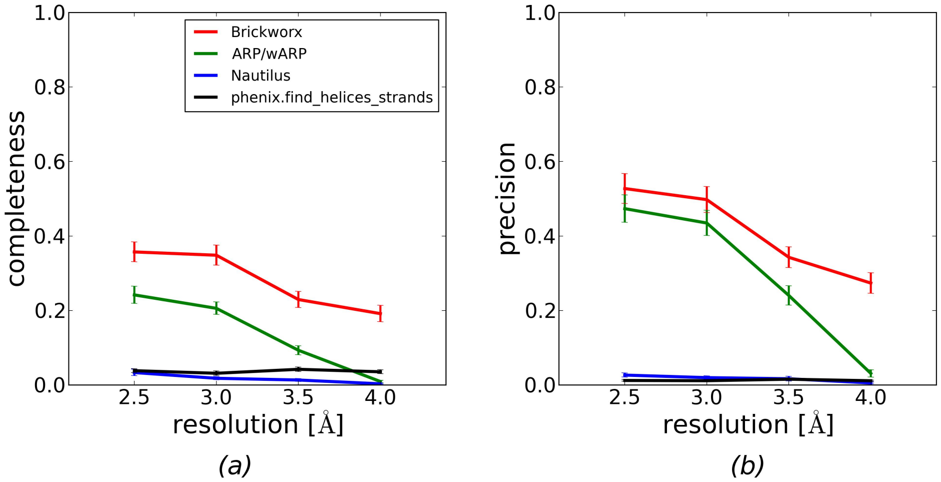

Figure S29: Completeness (a) and precision (b) of the model building algorithms implemented in Brickworx, ARP/wARP, and Nautilus (red, green, and blue lines frespectively). The models quality was evaluated based on strict criterion (including base type and position). The results presented on the figures are based on maps calculated for the protein-DNA complexes with mean phase error and figure-of-merit of $\langle|\Delta\phi|\rangle = 35°$ and $\langle m \rangle = 0.75$ respectively.

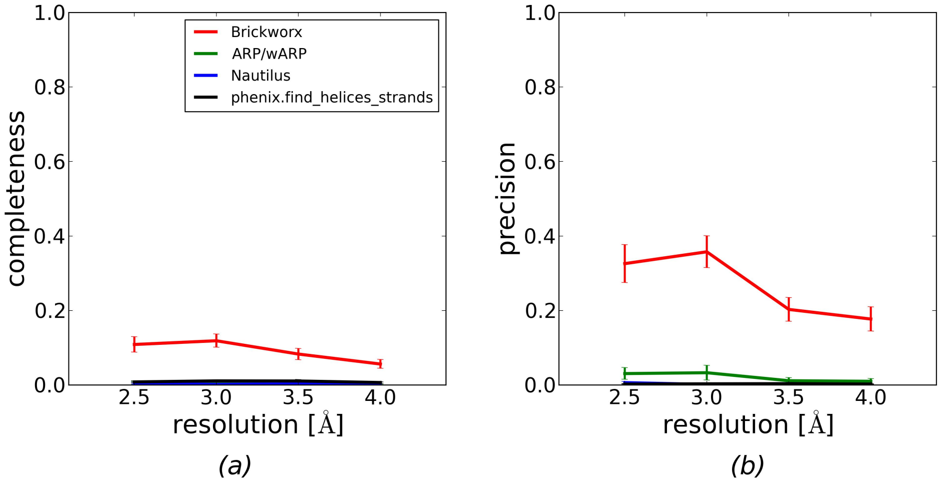

Figure S30: Completeness (a) and precision (b) of the model building algorithms implemented in Brickworx, ARP/wARP, and Nautilus (red, green, and blue lines frespectively). The models quality was evaluated based on strict criterion (including base type and position). The results presented on the figures are based on maps calculated for the protein-DNA complexes with mean phase error and figure-of-merit of $\langle|\Delta\phi|\rangle = 54°$ and $\langle m \rangle = 0.50$ respectively.

2.7 protein-RNA complexes (backbone position evaluated)

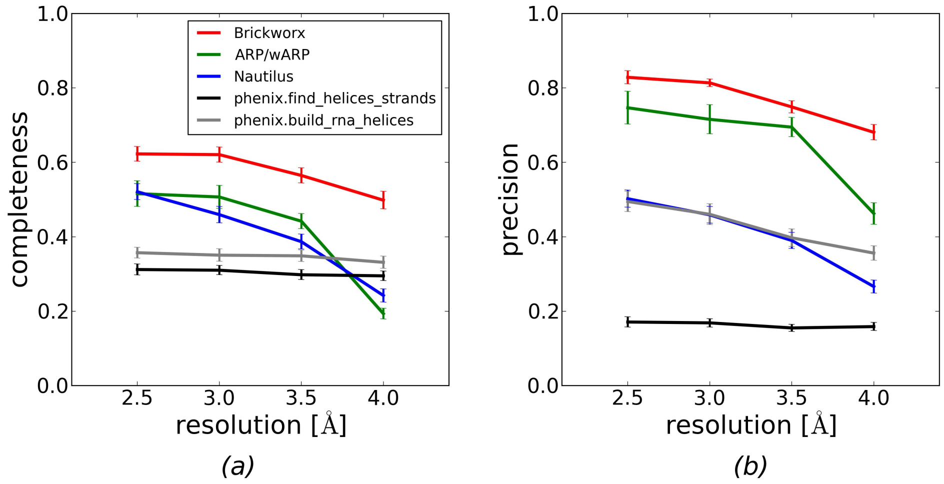

Figure S31: Completeness (a) and precision (b) of the model building algorithms implemented in Brickworx, ARP/wARP, and Nautilus (red, green, and blue lines frespectively). Only phosphorus and $C1'$ atom position were evaluated in the output models. The results presented on the figures are based on maps calculated for the protein-RNA complexes with mean phase error and figure-of-merit of $\langle|\Delta\phi|\rangle = 18°$ and $\langle m\rangle = 0.92$ respectively.

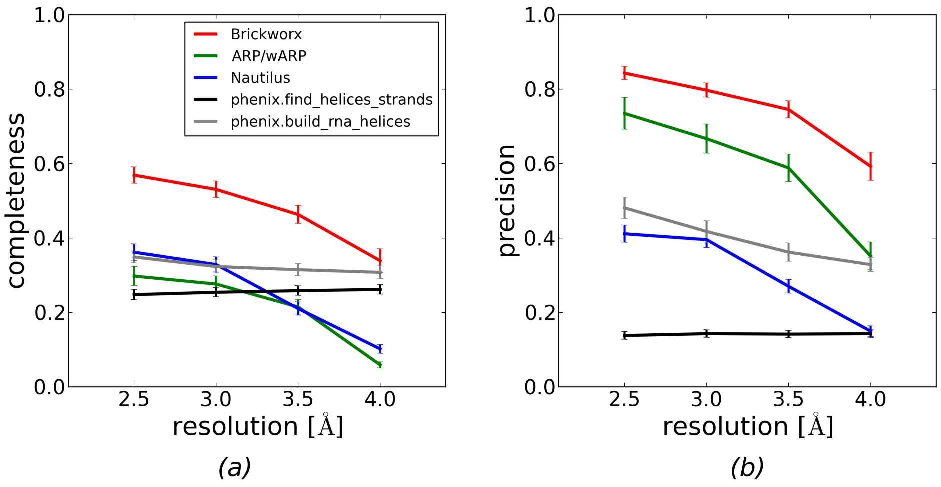

Figure S32: Completeness (a) and precision (b) of the model building algorithms implemented in Brickworx, ARP/wARP, and Nautilus (red, green, and blue lines frespectively). Only phosphorus and $C1'$ atom position were evaluated in the output models. The results presented on the figures are based on maps calculated for the protein-RNA complexes with mean phase error and figure-of-merit of $\langle|\Delta\phi|\rangle = 35°$ and $\langle m \rangle = 0.75$ respectively.

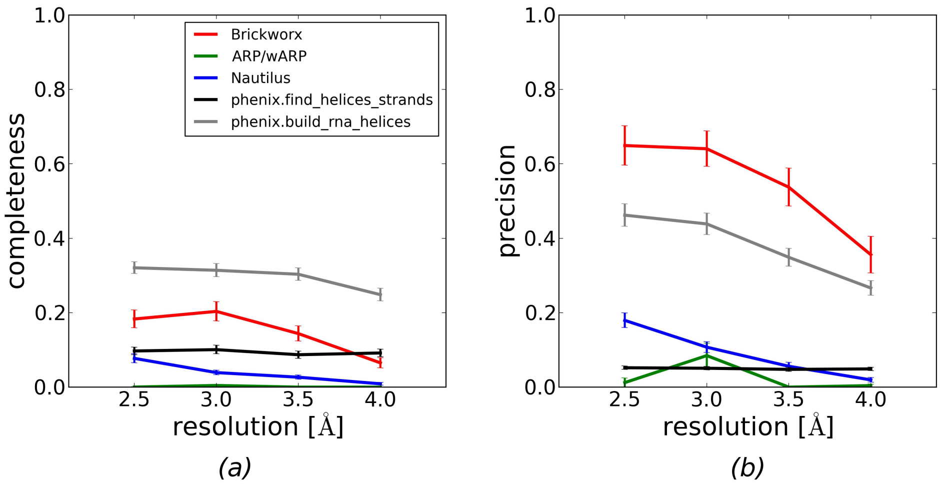

Figure S33: Completeness (a) and precision (b) of the model building algorithms implemented in Brickworx, ARP/wARP, and Nautilus (red, green, and blue lines frespectively). Only phosphorus and $C1'$ atom position were evaluated in the output models. The results presented on the figures are based on maps calculated for the protein-RNA complexes with mean phase error and figure-of-merit of $\langle|\Delta\phi|\rangle = 54°$ and $\langle m \rangle = 0.50$ respectively.

2.8 protein-RNA complexes (base type and postion evaluated)

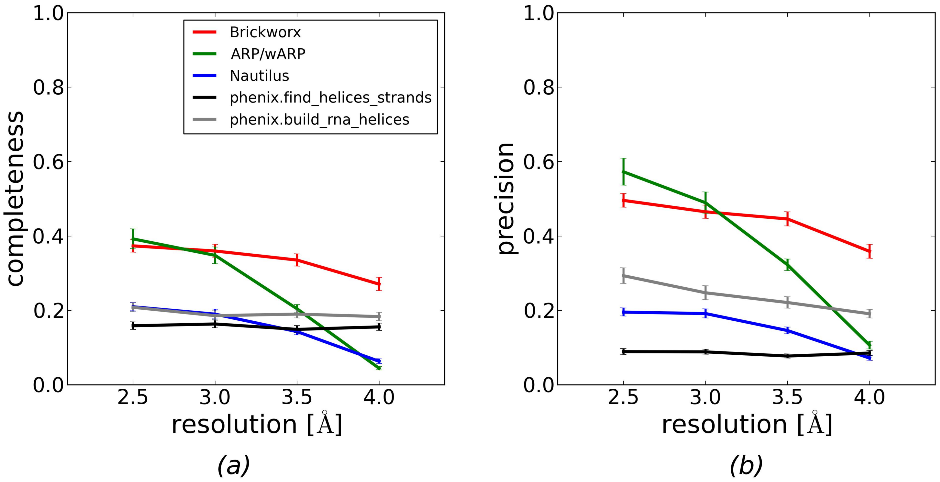

Figure S34: Completeness (a) and precision (b) of the model building algorithms implemented in Brickworx, ARP/wARP, and Nautilus (red, green, and blue lines frespectively). The models quality was evaluated based on strict criterion (including base type and position). The results presented on the figures are based on maps calculated for the protein-RNA complexes with mean phase error and figure-of-merit of $\langle|\Delta\phi|\rangle = 18°$ and $\langle m \rangle = 0.92$ respectively.

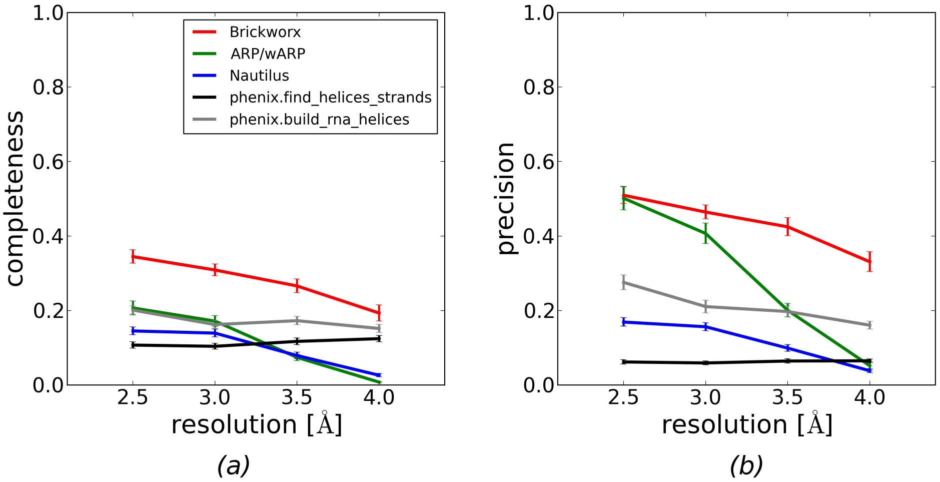

Figure S35: Completeness (a) and precision (b) of the model building algorithms implemented in Brickworx, ARP/wARP, and Nautilus (red, green, and blue lines frespectively). The models quality was evaluated based on strict criterion (including base type and position). The results presented on the figures are based on maps calculated for the protein-RNA complexes with mean phase error and figure-of-merit of $\langle|\Delta\phi|\rangle = 35°$ and $\langle m \rangle = 0.75$ respectively.

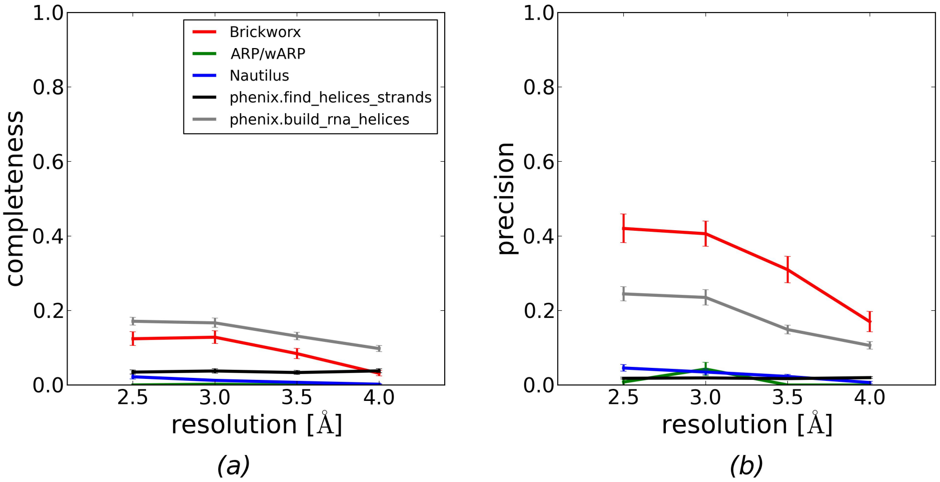

Figure S36: Completeness (a) and precision (b) of the model building algorithms implemented in Brickworx, ARP/wARP, and Nautilus (red, green, and blue lines frespectively). The models quality was evaluated based on strict criterion (including base type and position). The results presented on the figures are based on maps calculated for the protein-RNA complexes with mean phase error and figure-of-merit of $\langle|\Delta\phi|\rangle = 54°$ and $\langle m \rangle = 0.50$ respectively.

3 Brickworx computation-time benchmarks

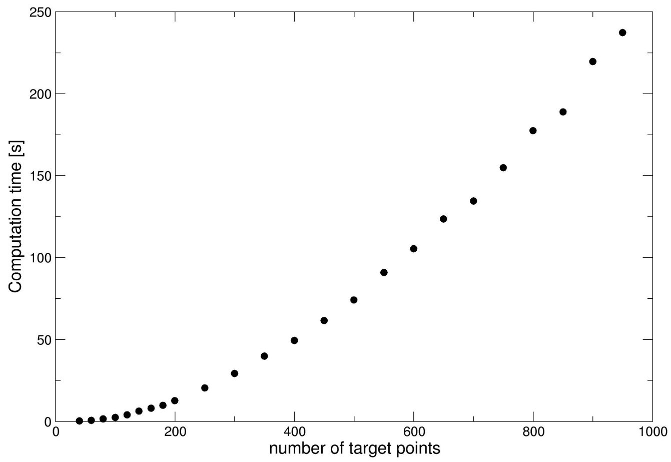

Figure S37: Average computation time of the Brickworx algorithm for matching nucleic acid fragments. In the simulations a 4 base-pairs A-RNA double helix was matched to random target patterns of phosphorus atoms.

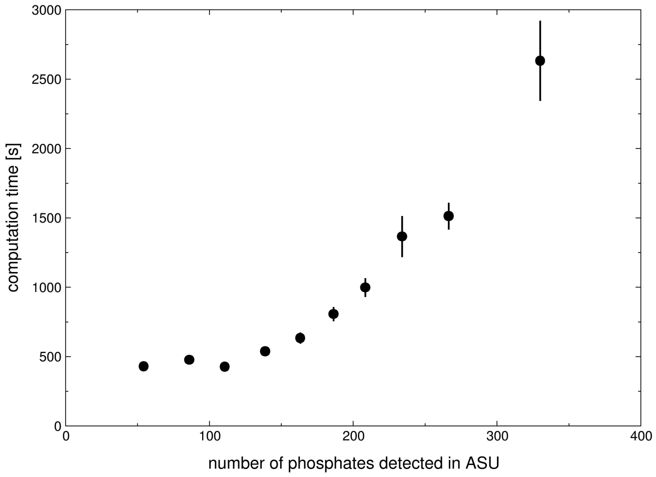

Figure S38: Average Brickworx computation time as a function of number of deetcted phosphorus atoms. Calculations were perfomed for all the testset RNA-containing structures.

4 Matching algorithm computational complexity estimates

Definition 1 *Let P be a set of n points in Euclidean 3-dimensional space R^3. The $C(n)$ is the maximum possible number of pairs of points in P with distance exactly 1.*

The exact formula for $C(n)$ is not known, however there exists some non-trivial bounds.

There are constructions of set P such that there are at least $n^{4/3} \log\log n$ pairs of points with distance 1.

Theorem 1 *[1]* $C(n) = \Omega(n^{4/3} \log\log n)$

And it can be proved that there are at most $n^{3/2}$ of such pairs.

Theorem 2 *[2]* $C(n) = O(n^{3/2})$

Please note, that theorems 1 and 2 consider worst-case-scenario or specially crafted data. For set of points that usually appear in the data the number of pairs with distance 1 seems to be sub-linear.

Algorithm 1: MATCHING(M, P, ϵ, r)

Input: Motif M and target pattern P – set of points in R^3,

$0 \leq \epsilon$ – error tolerance bound,

$0 \leq r$ – bound on RMSD

Output: Returns False if M does not occur in P or set of all matchings $g : M \to P$ with RMSD $\leq r$

```
Y = ∅
foreach (m1, m2, m3) ∈ M do
    foreach (p1, p2) ∈ P such that d(p1, p2) − d(m1, m2) ≤ ε do
        foreach p3 ∈ P such that d(p1, p3) − d(m1, m3) ≤ ε and
        d(p2, p3) − d(m2, m3) ≤ ε do
            compute rotation and translation that transforms (m1, m2, m3)
            into (p1, p2, p3)
            apply transformation to all points from M obtaining M′
            for each point from M′ find O(1) closest neighbors from P
            compute maximal cardinality matching g from M′ to P′ ⊆ P
            if |g| ≥ 3 and RMSD(M′, P′) ≤ r then
                Y = Y + {g}
if Y ≠ ∅ then
    return Y
else
    return False
```

The time complexity of the algorithm depends on the choice of ϵ.

If $\epsilon = 0$:

- there are most $|P|^{3/2}$ pairs (p_1, p_2) with distance $d(m_1, m_2)$ (we can use theorem 2),
- there are $O(|M|^3)$ candidates for (m_1, m_2, m_3) and $O(|P|)$ candidates p_3.
- computation of the transformation takes $O(1)$ time since we transform sets of 3 points.
- Obtaining M' and localization of its neighbours can be done in $O(|M| \cdot \max(\sqrt{|M|}, \log |P|))$.

This will give the $O(|M|^3|P|^{5/2} \cdot |M| \cdot \max(\sqrt{|M|}, \log |P|)$ time. In practice such high complexity is never observed and the running time can be reduced almost to:

$$O((\#\text{number of congruent triangles in M and P}) \cdot |M| \cdot \max(\sqrt{|M|}, \log |P|).$$

If $\epsilon = \infty$ we have to consider $|M|^3$ triples (m_1, m_2, m_3) and $|P|^3$ triples (p_1, p_2, p_3), so this will result in the $O(|M|^3|P|^3 \cdot |M| \cdot \max(\sqrt{|M|}, \log |P|)$ time complexity.

References

[1] P. Erdös. *On sets of distances on n points in euclidean space.* 5:165–169, 1960.

[2] Haim Kaplan, Jirí Matousek, Zuzana Safernová, and Micha Sharir. *Unit distances in three dimensions* Combinatorics, Probability & Computing, 21(4):597–610, 2012.

5 Benchmarks with experimantal electron-denity maps

Method	Total number of nucleotides built	Correctly placed nucleotides	Nucleotides with a correct base-type
BrickworX	233	179	119
BrickworX - RNA stems only	167	118	73
ARP/wARP	234	84	25
Nautilus	131	67	21
phenix.find_helices_strands	194	56	25
phenix.build_rna_helices	114	72	45

Table S1: Validation of Group II intron (PDB: 3bwp) models built using various methods. The reference structure contains 388 RNA nucleotides in total.

Method	Total number of nucleotides built	Correctly placed nucleotides	Nucleotides with a correct base-type
BrickworX	51	41	13
BrickworX - RNA stems only	36	29	8
ARP/wARP	0	0	0
Nautilus	31	4	2
phenix.find_helices_strands	94	32	12
phenix.build_rna_helices	64	37	18

Table S2: Validation of Lysine riboswitch (PDB: 3d0u) models built using various methods. The reference structure contains 161 RNA nucleotides in total.

6 Support Vector Machine classifier training set structures

4kzx, 1fjg, 2rkj, 3w1k, 3g8s, 4lck, 1kog, 2xxa, 3kfu, 3iwn, 3ktw, 1qzw, 3ivk, 3wfq, 1u6b, 1wz2, 3p49, 2zni, 1efw, 3cun, 2nr0, 3w3s, 3epj, 3a2k, 3ndb, 1un6, 1mfq, 4ilm, 2d6f, 3wc1, 4o26, 2der, 3mur, 1u63, 2xd0, 1j2b, 3icq, 2nqp, 3b0v, 2bny, 3amu, 2du5, 1ob2, 4kr7, 3dh3, 2du6, 1eiy, 1h4q, 4kr2, 3qsy, 1euq, 3al0, 3ov7, 2ake, 1vc5, 3oij, 4c9d, 4ang, 4kji, 1q2s, 3id5, 3uzs, 1zbh, 4gcw, 1ze2, 2nre, 1xok, 2r93, 2b63, 2zh7

7 Reference structures used for benchmarks

1. **DNA**
   116d, 1d67, 1d7z, 1ei4, 1fdg, 1fn2, 1i0o, 1k2l, 1p4y, 1p4z, 1qv4, 1yb9, 1z5t, 1zf3, 1zf4, 1zf7, 1zfe, 1zph, 1zpi, 233d, 249d, 263d, 265d, 270d, 285d, 298d, 2b0k, 2b1d, 2b2b, 2b3e, 2d25, 2i2i, 2nsk, 2p8d, 2pl8, 2plb, 311d, 334d, 345d, 348d, 360d, 3oie, 3qf8, 3sd8, 3ukb, 3uyb, 3v9d, 401d, 436d, 459d

2. **protein-DNA complexes**
   1wd1, 2bcs, 1b96, 1r71, 2h1k, 1wbb, 2nq9, 2jg3, 2evh, 1rxw, 2e52, 1l5u, 1u48, 1je8, 2r8i, 2ram, 1krp, 2noi, 2vih, 1skr, 1l2d, 2a07, 1zet, 1ztw, 2owo, 1ua1, 1lq1, 2c6y, 2a66, 1rm1, 2ezv, 2fmq, 1w7a, 2dtu, 2gws, 2p0j, 2uz4, 1ua0, 1njw, 1sl2, 2gig, 2f5n, 1a6y, 1wb9, 2nqj, 2vy1, 1pp7, 2cgp, 2imw, 1d3u

3. **RNA**
   1hr2, 3pdr, 2gdi, 1et4, 3rg5, 3d2g, 3dil, 4p95, 2qus, 3ski, 3gx5, 3p22, 1q93, 2hoj, 4k27, 3sd3, 1y27, 4fnj, 1y3s, 2gcs, 3e5c, 1ehz, 3la5, 3nkb, 1y26, 2zy6, 1msy, 2oeu, 4jf2, 3b31, 3szx

4. **Protein-RNA complexes**
   2vqe, 2nz4, 2v3c, 2y9h, 2qux, 4oo8, 3rw6, 3foz, 3add, 1g59, 2vpl, 4m4o, 1mji, 4aq7, 1m5k, 3u4m, 1qtq, 3am1, 1j1u, 1wsu, 2i82, 1jid, 1u0b, 2b3j, 2zue, 1qu2, 2bu1, 1f7u, 1hq1, 3egz, 1c0a, 1jbs, 4m30, 3hjw, 3ts2, 2ez6, 4ill, 4bw0, 1a9n, 1cx0, 2hw8, 2pjp, 2xlk, 1jbr, 3vjr, 4l8h, 3bt7, 2ann, 4c8z, 4oog, 4al5, 2xli, 2y8w, 3ova, 1urn, 2f8k, 2y8y, 1k8w, 3oin, 1r3e, 2dr8, 1c9s
